# *Ent*-Abietanoids Isolated from *Isodon serra*

**DOI:** 10.3390/molecules22020309

**Published:** 2017-02-17

**Authors:** Jun Wan, Hua-Yi Jiang, Jian-Wei Tang, Xing-Ren Li, Xue Du, Yan Li, Han-Dong Sun, Jian-Xin Pu

**Affiliations:** 1 State Key Laboratory of Phytochemistry and Plant Resources in West China, Kunming Institute of Botany, Chinese Academy of Sciences, Kunming 650201, China; wanjun@imm.ac.cn (J.W.); jianghuayi@mail.kib.ac.cn (H.-Y.J.); tangjianwei@mail.kib.ac.cn (J.-W.T.); lixingren@mail.kib.ac.cn (X.-R.L.); duxue@mail.kib.ac.cn (X.D.); liyanb@mail.kib.ac.cn (Y.L.); hdsun@mail.kib.ac.cn (H.-D.S.); 2 Kunming College of Life Sciences, University of Chinese Academy of Sciences, Beijing 100039, China

**Keywords:** *Isodon serra*, *ent*-abietane diterpenoids, anti-inflammation, cytotoxicity

## Abstract

Four new *ent*-abietane diterpenoids, along with four known ones were isolated from the aerial parts of *Isodon serra*, a traditional Chinese folk medicine. The new diterpenoids were named as serrin K (**1**), xerophilusin XVII (**2**), and enanderianins Q and R (**3** and **4**), while the known ones were identified as rubescansin J (**5**), (3α,14β)-3,18-[(1-methylethane-1,1-diyl)dioxy]-*ent*-abieta-7,15(17)-diene-14,16-diol (**6**), xerophilusin XIV (**7**), and enanderianin P (**8**), respectively. Their structures were elucidated by extensive spectroscopic analysis and comparison with the literature. Compound **1** showed remarkable inhibitory activity towards NO production in LPS-stimulated RAW264.7 cells (IC_50_ = 1.8 μM) and weak cytotoxicity towards five human tumor cell lines (HL-60, SMMC-7721, A-549, MCF-7, SW480).

## 1. Introduction

**Figure 1:**
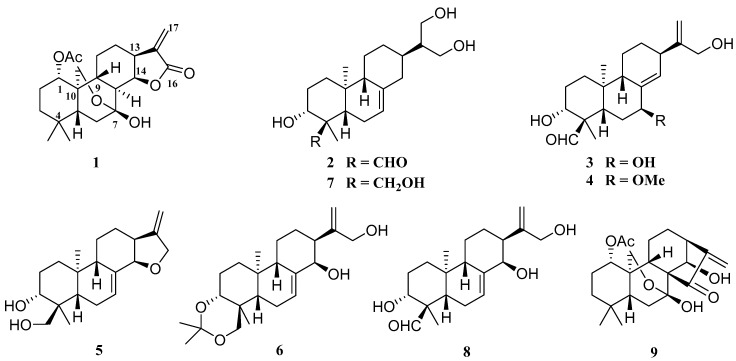
*Ent*-abietanoids (**1**–**8**) isolated from *Isodon serra* and rabdocoestin B (**9**).

The perennial plant *Isodon serra* (Maxim.) Hara, belonging to the Lamiaceae, has long been used as Chinese folk medicine for the treatment of jaundice hepatitis, acute cholecystitis, and enteritis [1,2]. People in the Guangdong Province of China have been processing this herb into tea bags and granules which are used to protect the liver and cholecyst [3]. Previous phytochemical investigations of this species have afforded abundant bioactive *ent*-kaurane diterpenoids [4,5,6,7,8,9,10]. Our continuing research for bioactive constituents of the aerial parts of this plant collected in the E′mei mountain, Sichuan Province of China, has led to the discovery of eight diterpenoids, including four new *ent*-abietanoids, named serrin K (**1**), xerophilusin XVII (**2**), and enanderianins Q and R (**3** and **4**), together with four known analogues, rubescansin J (**5**), (3α,14β)-3,18-[(1-methylethane-1,1-diyl)dioxy]-*ent*-abieta-7,15(17)-diene-14,16-diol (**6**) [11], xerophilusin XIV (**7**) [12], and enanderianin P (**8**) [13] (Figure 1). In contrast to the previous chemical investigations of this plant, this was the first discovery of the *ent*-abietane diterpenoids from this species. Due to the excellent anti-tumor and anti-inflammatory effects of diterpenoids isolated from *I. serra* [14,15,16,17,18,19,20,21,22] and its wide use towards hepatitis and cholecystitis in Chinese folk medicine, some of the isolates have been assayed for their anti-tumor effects against five human tumor cell lines (HL-60, SMMC-7721, A-549, MCF-7, SW480), as well as the inhibitory activity of NO production in LPS-stimulated RAW264.7 cells. Among them, compound **1** showed weak cytotoxicity, but remarkable NO production inhibitory activity (IC_50_ = 1.8 μM). Reported herein are the isolation, structure elucidation, and the biological evaluation of the above-mentioned compounds.

## 2. Results and Discussion

The air-dried aerial parts of *I. serra* were extracted with 70% aqueous acetone solution (*v*/*v*), yielding crude extracts, which then were partitioned between EtOAc and H_2_O. The EtOAc-solubles were subjected to repeated column chromatography and then HPLC to afford four new *ent*-abietane diterpenoids named serrin K (**1**), xerophilusin XVII (**2**), and enanderianins Q and R (**3** and **4**).

**Figure 2:**
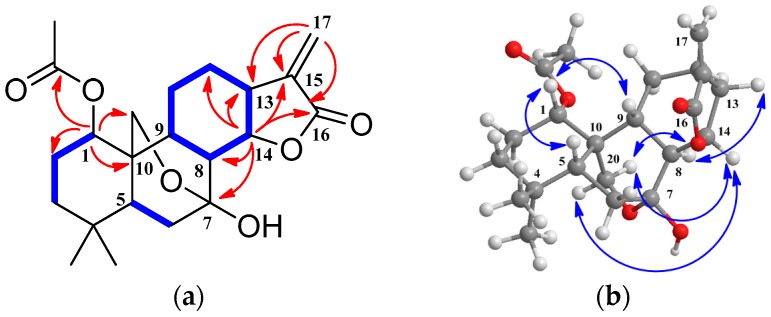
The 2D NMR correlations of compound **1**: (**a**) ^1^H-^1^H correlation spectroscopy (^1^H-^1^H COSY) (bold) and selected heteronuclear multiple bond correlations (HMBC) (arrows); (**b**) The rotating-frame overhauser effect spectroscopy (ROESY) correlations.

Compound **1** was obtained as a white amorphous powder. The high resolution electrospray ionization mass spectroscopy (HRESIMS) (Figure S4) of **1** exhibited a [M + Na]^+^ peak at 413.1942, which suggested a molecular formula of C_22_H_30_O_6_ (M = 390.2042), indicating eight degrees of unsaturation. Its IR spectrum had absorption bands at 3439, 1760, 1736, and 1632 cm^−1^, accounting for the presence of hydroxyl, carbonyl, and disubstituted olefinic groups. The ^1^H-NMR (Table 1 and Figure S1) showed three singlet methyl signals, among which, 0.74 and 1.07 were attributed to H_3_-18 and H_3_-19, and 2.07 belonged to OAc. The signals at 4.21 and 4.35 (each 1H, d, *J* = 10.3 Hz) indicated an AB characteristic oxygenated methylene, which might be attributed to H_2_-20. The ^13^C-NMR spectrum (Table 2 and Figure S1) exhibited 22 carbon signals, including three methyls, seven methylenes (one oxygenated, one exocyclic-olefinic), six methines (two oxygenated) and six quaternary carbons (one oxygenated, and two carbonyl carbons). Despite differences in certain chemical shifts, the NMR spectroscopic data of **1** resembled that of rabdocoestin B (**9**) [23] (Figure 1), suggesting that the A–C rings of **1** and **9** were extremely similar, whereas the D-ring displayed a *γ*-lactone structure established after the carbonyl signal up-field shift (*δ*_C_ 170.8 of **1** compared to *δ*_C_ 204.4 of **9**), the down-field shift of H-C-(14)-O (*δ*_H_ 5.31 of **1** compared to *δ*_H_ 5.04 of **9**), and the replacement of one quaternary carbon (*δ*_C_ 59.7, s) in **9** by a methine (*δ*_C_ 45.4, d) in **1**. The structure of **1** was supported by 2D NMR experiments. The ^1^H-^1^H correlation spectroscopy (^1^H-^1^H COSY) spectrum (Figure S2) showed correlations of H-5/H_2_-6 and H-8/H-9/H_2_-11/H_2_-12/H-13/H-14/H-8, along with the heteronuclear multiple bond correlations (HMBC) from H-14 (*δ*_H_ 5.31, t) to C-7 (*δ*_C_ 96.8, s), C-8 (*δ*_C_ 45.4, d), C-12 (*δ*_C_ 27.7, t) , C-13 (*δ*_C_ 39.5, d), C-15 (*δ*_C_ 140.9, s), and C-16 (*δ*_C_ 170.8, s), and from H_2_-17 (*δ*_H_ 5.52, 6.33) to C-13, C-15, and C-16, indicating an ester group and thus completing the D-ring as a *γ*-lactone ring with an exocyclic double bond (Figure 2). ^1^H-^1^H COSY correlations of H-1/H_2_-2/H_2_-3 combined with the HMBC from H_2_-2 (*δ*_H_ 1.55, 1.80), H_2_-3 (*δ*_H_ 1.17, 1.32), H_2_-20 (*δ*_H_ 4.21, 4.35), and H_3_-OAc (*δ*_H_ 2.07) to C-1 (*δ*_C_ 76.5, d), and from H-1 (*δ*_H_ 4.81) to C-2 (*δ*_C_ 25.8, t), C-9 (*δ*_C_ 47.0, d), C-10 (*δ*_C_ 37.7, s), C-20 (*δ*_C_ 64.1, t), and OAc (*δ*_C_ 170.6, s) revealed an OAc group at C-1. The rotating-frame overhauser effect spectroscopy (ROESY) spectrum (Figure S3) showed correlations of H-1/H-5/H-9, H-13/H-8, and H-14/H_2_-20, illustrating the β-, α-*,* and α-orientation of H-1, H-8, and H-14, respectively, as well as the β-orientation of the D-ring (Figure 2). Thus, the structure of compound **1** was determined as 1α-acetoxy-7α,20-epoxy-7β-hydroxy-*ent*-abieta-15(17)-en-16,14β-lactone, and it was named serrin K.

Compound **2**, obtained as a light yellow powder, displayed an ion peak [M + Na]^+^ at 359.2186 (calcd. 359.2193), corresponding to the molecular formula C_20_H_32_O_4_. Compound **2** had an analogue chemical structure to xerophilusin XIV (**7**) [12] (Figure 1) accounting for their similar NMR spectra, except for the absence of a hydroxymethyl (*δ*_C_ 67.5, t) in **7** and the presence of one formyl group (*δ*_C_ 207.3, d) in **2**. The ^1^H-^1^H COSY correlations of H_2_-1/H_2_-2/H-3, along with the HMBC from H-CHO (*δ*_H_ 9.62, s) to C-4 (*δ*_C_ 56.2, s), Me (*δ*_C_ 10.3, q), and C-3 (*δ*_C_ 72.8, d), and from H-3 (*δ*_H_ 4.12) to C-4 (*δ*_C_ 56.2, s), Me (*δ*_C_ 10.3, q), and C-CHO (*δ*_C_ 207.3, d), revealed that C-3 was substituted by an OH group and C-18 was replaced by a formyl group. The ROESY correlations of H-3/H-5/H_3_-18, along with the up-field shift of C-5 (Δ*δ* 7.2 ppm) compared with serrin K (**1**) due to the *γ*-gauche steric compression effect between 18-CHO and H-5β, indicating the OH and the formyl group were α- and β-orientation, respectively. Therefore, the structure of **2** was elucidated as 3α,16,17-trihydroxy-*ent*-abieta-7-en-18-al, and it was named xerophilusin XVII.

**Figure 3:**
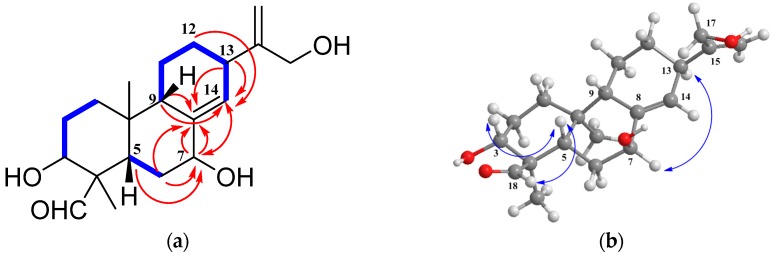
The 2D NMR correlations of compound **3**: (**a**) The ^1^H-^1^H COSY (bold) and selected HMBC (arrows) correlations; (**b**) The ROESY correlations.

Compound **3** had the same molecular formula as enanderianin P (**8**) [13], C_20_H_30_O_4_, on the basis of HRESIMS. NMR data comparison of the two compounds suggested that they were structurally similar with only the position changes of the hydroxy and endocyclic olefinic groups in **3**. ^1^H-^1^H COSY correlations of H-5/H_2_-6/H-7, together with the HMBC from H-5 (*δ*_H_ 2.61), H-6b (*δ*_H_ 1.57), and H-14 (*δ*_H_ 5.86) to C-7 (*δ*_C_ 71.9, d), indicated an OH group at C-7 in **3** (Figure 3). The ^1^H-^1^H COSY correlations of H-9/H_2_-11/H_2_-12/H-13/H-14, along with the HMBC from H_2_-6 (*δ*_H_ 1.78, 1.57), H-7 (*δ*_H_ 4.40), H-9 (*δ*_H_ 2.64), and H-13 (*δ*_H_ 2.89) to C-8 (*δ*_C_ 141.3, s), and from H-7, H-9, H_2_-12 (*δ*_H_ 1.92, 1.37), and H-13 to C-14 (*δ*_C_ 129.3, d), disclosed an endocyclic double bond between C-8 and C-14. The *β*-orientation of HO-7 was deduced by the H-7/H-13 ROESY correlation (Figure 3). Hence, the structure of **3** was determined as 3α,7β,16-trihydroxy-*ent*-abieta-8(14),15(17)-diene-18-al, and it was named enanderianin Q.

HRESIMS established the molecular formula of compound **4** as C_21_H_32_O_4_ with six degrees of unsaturation. Preliminary inspection of the NMR spectra of **4** suggested that it resembled **3**, except for displaying one methoxy group (*δ*_C_ 54.8, q). The 2D NMR spectra showed HMBC correlations from MeO (*δ*_H_ 3.16) to C-7 (*δ*_C_ 81.3, d), revealing a MeO substituent at C-7. ROESY correlations disclosed that the orientations of the substituents in **4** were the same as those of **3**. Therefore, the structure of **4** was determined to be 3α,16-dihydroxy-7β-methoxy-*ent*-abieta-8(14),15(17)-dien-18-al, and it was named enanderianin R.

Compounds **5**–**8** were identified by comparing their physical constant data with those reported in the literature. All of the above compounds are *ent*-abietane type diterpenoids, which are different from the previous diterpenoid types isolated from this species. Previous studies showed that the *ent*-kauranoids exhibited potent anti-tumor activities [3,24]. Therefore, with the aim of exploring diterpenoids with anti-tumor and anti-inflammatory activity which might be related to the wide use of *I. serra* in Chinese folk medicine, compounds with qualified sample quantity (>1 mg), **1** and **3**–**8**, were subjected to the assay for cytotoxic activity against five human tumor cell lines (HL-60, SMMC-7721, A-549, MCF-7, SW480) and for the inhibitory activity of NO production in LPS-stimulated RAW264.7 cells. Among these, only compound **1** showed some cytotoxic potency (IC_50_ ranging from 9.4 to 20.4 μM), while compounds **3**–**8** exhibited no cytotoxicity (positive control, *cis*-platin, IC_50_ ranging from 1.9–18.3 μM). Moreover, compound **1** also revealed significant NO production inhibitory activity with an IC_50_ of 1.8 μM (positive control, MG132, IC_50_ = 0.2 μM).

## 3. Experimental Section

### 3.1. General Information

Optical rotations were measured in MeOH on a JASCO P-1020 digital Polarimeter, whereas UV spectra data were obtained on a Shimadzu UV2401PC spectrophotometer (Shimadzu, Kyoto, Japan). A Tensor 27 spectrophotometer (Bruker, Karlsruhe, Germany) was used for scanning IR spectroscopy with KBr pellets. 1D- and 2D-NMR (*δ*_H_ 8.71, 7.55 and 7.19 for pyridine-*d*_5_) spectra were recorded on Bruker AM-400, DRX-500, and DRX-600 spectrometers (Bruker Biospin, Zurich, Switzerland). Unless otherwise specified, chemical shifts (*δ*) were expressed in ppm with reference to the solvent signals. HRESIMS was performed on a VG Autospec-3000 spectrometer (VG Instruments, UK) at 70 eV. Column chromatography (CC) was performed with silica gel (100–200 mesh; Qingdao Marine Chemical, Inc., Qingdao, China), Sephadex LH-20 gel (40–70 μM, Amersham Pharmacia Biotech AB, Uppsala, Sweden), and Lichroprep RP-18 gel (40–63 μM, Merck, Darmstadt, Germany). Semi-preparative HPLC was performed on Agilent 1100 and Agilent 1200 liquid chromatographs (Agilent Technologies, Santa Clara, CA, USA) with a Zorbax SB-C18 (9.4 mm × 25 cm) column. Preparative HPLC was performed on an Agilent 1260 liquid chromatograph with a Zorbax SB-C18, 21 mm × 25 cm column. Fractions were decolored on MCI gel (75–150 μM, Mitsubishi Chemical Corporation, Tokyo, Japan), and monitored by TLC. The spots were visualized by heating silica gel plates sprayed with 5% H_2_SO_4_ in EtOH. All solvents including petroleum ether were distilled prior to use.

### 3.2. Plant Material

The aerial parts of *I. serra* were collected in the E’mei Mountain, Sichuan Province, Leshan, China, in August 2008. The voucher specimen (KIB 2008091703) was deposited at the State Key Laboratory of Phytochemistry and Plant Resources in West China, Kunming Institute of Botany, Chinese Academy of Sciences, and was identified by Prof. Xi-Wen Li.

### 3.3. Extraction and Isolation

The air-dried aerial parts (ca. 6.0 kg) of *I. serra* were powdered and extracted with acetone–H_2_O (3 × 20 L, 70:30, *v*/*v*, each for 3 days) at room temperature to produce a crude extract. The extracts were combined and concentrated to about a 2 L water layer which was successively partitioned by EtOAc (5 × 2 L), resulting in EtOAc extract (ca. 300.0 g). This portion was then subjected to silica gel CC (1 kg, 100–200 mesh), eluting with CHCl_3_–Me_2_CO (1:0 → 9:1 → 8:2 → 7:3 → 6:4 → 5:5 → 0:1 gradient system, 35 L for each) to produce seven fractions (Fr. 1–7). Each fraction was then decolorized on MCI gel, and eluted with MeOH–H_2_O (90:10, *v*/*v*, 8 L for each).

Fr. 2 (crude crystals, 14.0 g, CHCl_3_–Me_2_CO 9:1) was then washed repeatedly with MeOH to obtain a soluble-part (ca. 3.2 g), which afterwards was divided into 6 sub-fractions (Fr. 2-1-1 to Fr.2-1-6) by chromatography on Sephadex LH-20 gel (CHCl_3_–MeOH 1:1 *v*/*v*, 1.5 L). Among which, Fr. 2-1-4 (280 mg) was subjected to preparative HPLC (CH_3_CN–H_2_O, 45:55 *v*/*v*, 15 mL/min, 2.8 L) to yield four parts (Fr. 2-1-4-1 to Fr. 2-1-4-4). Subfraction Fr. 2-1-4-2 (52 mg) was then subjected to semi-preparative HPLC (CH_3_CN–H_2_O, 30:70 *v*/*v*, 3 mL/min, 2.3 L) to produce compound **1** (30.0 mg).

Fr. 3 (37.0 g, CHCl_3_–Me_2_CO 8:2) was subjected to a RP-18 CC eluted with MeOH–H_2_O gradient (30:70 → 40:60 → 50:50 → 60:40 → 70:30 → 80:20 → 90:10 → 100:0 *v*/*v*, each gradient eluted for 10 L) to yield subfractions Fr. 3-1 to Fr. 3-8. Fr. 3-3 (2.2 g, MeOH–H_2_O 50:50 *v*/*v*) was chromatographed on Sephadex LH-20 gel (CHCl_3_–MeOH 1:1 *v*/*v*, 1.2 L) to yield 4 parts (Fr. 3-3-1 to Fr. 3-3-4), and the main part, Fr. 3-3-3 (1.4 g), was then applied to a RP-18 CC (MeOH–H_2_O, 45:55 *v*/*v*, 5 L) to be divided into 11 sub-fractions (Fr. 3-3-3-1 to Fr. 3-3-3-11). Fr. 3-3-3-8 (36.0 mg) was subjected to semi-preparative HPLC (CH_3_CN–H_2_O, 32:68 *v*/*v*, 3 L) to obtain compound **5** (13.2 mg) and compound **6** (3.0 mg). Fr. 3-5 (30.0 g, crude crystals, MeOH–H_2_O 70:30) was washed with MeOH repeatedly to give a corresponding solution, Fr. 3-3-1 (8.436 g), which was then separated by silica gel (200 mesh, CHCl_3_–Me_2_CO, gradient 20:1 → 10:1 → 8:2 → 7:3 → 6:4 → 1:1 *v*/*v*, each 2 L) to produce six portions (Fr. 3-5-1-1 to Fr. 3-5-1-6). Fr. 3-5-1-1 (185.0 mg) was applied to Sephadex LH-20 gel (CHCl_3_–MeOH 1:1, 0.3 L) and then by semi-preparative HPLC (CH_3_CN–H_2_O, 30:70 *v*/*v*, 3 mL/min, 1.8 L) to yield compound **2** (0.9 mg).

Fr. 4 (16.0 g, CHCl_3_–Me_2_CO 7:3) was subjected to a RP-18 CC (MeOH–H_2_O, 35:65 → 45:55 → 55:45 → 65:35 → 75:25 → 85:15 → 100:0 *v*/*v*) with monitoring by TLC (CHCl_3_:Me_2_CO 7:3 *v*/*v*) to yield 10 parts (Fr. 4-1 to Fr. 4-10), among which, Fr. 4-5 (3.8 g) was chromatographed on Sephadex LH-20 gel (CHCl_3_–MeOH 1:1, 1.5 L) to yield 6 sub-fractions. Fr. 4-5-6 (54.3 mg) was applied to semi-preparative HPLC (CH_3_CN–H_2_O, 27:73 *v*/*v*, 3 L) to yield compound **7** (6.4 mg). Fr. 4-7 (1.1 g) was chromatographed on Sephadex LH-20 gel (CHCl_3_–MeOH 1:1, 1 L), and one sub-fraction was subjected to preparative HPLC and then semi-preparative HPLC (CH_3_CN–H_2_O, 20:80 *v*/*v*, 3.8 L) to yield compounds **3** (5.3 mg), **4** (2.0 mg), and **8** (22.0 mg).

Compound **1**: White amorphous powder; [α${]}_{D}^{23}$ −48 (*c* 0.16, MeOH); UV (MeOH), *λ*_max_ 209 nm; IR (KBr), *ν*_max_ 3439, 2946, 1760, 1736, 1632, 1375, 1248, 1049 cm^−1^; HRESIMS (positive-ion mode) *m*/*z* 413.1942 [M + Na]^+^ (calcd. for C_22_H_30_O_6_Na, 413.1935); ^1^H-NMR (pyridine-*d*_5_, 600 MHz) and ^13^C-NMR (pyridine-*d*_5_, 150 MHz) spectra data, see Table 1 and Table 2.

Compound **2**: Light yellow amorphous powder; UV (MeOH), *λ*_max_ 204, 241 nm; IR (KBr), *ν*_max_ 3412, 2930, 1719, 1647, 1384, 1026 cm^−1^; HRESIMS (positive-ion mode) *m*/*z* 359.2186 [M + Na]^+^ (calcd. for C_20_H_32_O_4_Na, 359.2193); ^1^H-NMR (pyridine-*d*_5_, 600 MHz) and ^13^C-NMR (pyridine-*d*_5_, 150 MHz) spectra data, see Table 1 and Table 2.

Compound **3**: Light yellow amorphous powder; [α${]}_{D}^{25}$ +29 (c 0.15, MeOH); UV (MeOH), *λ*_max_ 203, 292 nm; IR (KBr), *ν*_max_ 3426, 2935, 1720, 1632, 1386, 1029, 599 cm^−1^; HRESIMS (positive-ion mode) *m*/*z* 357.2033 [M + Na]^+^ (calcd. for C_20_H_30_O_4_Na, 357.2036); ^1^H-NMR (pyridine-*d*_5_, 400 MHz) and ^13^C-NMR (pyridine-*d*_5_, 125 MHz) spectra data, see Table 1 and Table 2.

Compound **4**: Light yellow amorphous powder; [α${]}_{D}^{20}$ −104 (c 0.05, MeOH); UV (MeOH), *λ*_max_ 203, 283 nm; IR (KBr), *ν*_max_ 3427, 2935, 1724, 1642, 1385, 1047, 672 cm^−1^; HRESIMS (positive-ion mode) *m*/*z* 371.2188 [M + Na]^+^ (calcd. for C_21_H_32_O_4_Na, 371.2193); ^1^H-NMR (pyridine-*d*_5_, 500 MHz) and ^13^C-NMR (pyridine-*d*_5_, 125 MHz) spectra data, see Table 1 and Table 2.

Compound **5**: White amorphous powder; UV (MeOH), *λ*_max_ 204 nm; IR (KBr), *ν*_max_ 3418, 2935, 2863, 1733, 1712, 1559, 1226, 1071, 1031, 950 cm^−1^. HREIMS (positive-ion mode) *m*/*z* 318.2193 [M]^+^ (calcd. for C_20_H_30_O_3_, 318.2195); ^1^H-NMR (pyridine-*d*_5_, 400 MHz) *δ*: 1.79 and 1.18 (each 1H, m, H_2_-1), 1.89 and 1.62 (each 1H, m, H_2_-2), 4.17 (1H, dd, *J* = 10.8, 4.9 Hz, H-3β), 1.94 (1H, overlap, H-5β), 2.09–2.03 (2H, overlap, H_2_-6), 5.66 (1H, d, *J* = 2.1 Hz, H-7), 2.05 (1H, overlap, H-9β), 1.93 and 1.04 (each 1H, m, H_2_-11), 1.65 and 1.42 (each 1H, m, H_2_-12), 2.41 (1H, m, H-13α), 4.18 (1H, br. s, H-14α), 4.55 and 4.26 (each 1H, d, *J* = 14.0 Hz, H_2_-16), 5.01 and 4.84 (each 1H, br. s, H_2_-17), 4.06 and 3.60 (each 1H, d, *J* = 10.8 Hz, H_2_-18), 1.12 (Me, s, H_3_-19), 0.86 (Me, s, H_3_-20); ^13^C-NMR (pyridine-*d*_5_, 100 MHz) *δ*: 37.5 (t, C-1), 28.8 (t, C-2), 73.1 (d, C-3), 42.7 (s, C-4), 42.3 (d, C-5), 23.5 (t, C-6), 129.5 (d, C-7), 134.6 (s, C-8), 49.2 (d, C-9), 34.8 (s, C-10), 23.7 (t, C-11), 27.5 (t, C-12), 45.8 (d, C-13), 83.4 (d, C-14), 154.5 (s, C-15), 69.4 (t, C-16), 102.9 (t, C-17), 67.1 (t, C-18), 12.6 (q, C-19), 15.0 (q, C-20).

Compound **6** (acetonide derivative of rubescensin I): White amorphous powder; ^1^H-NMR (pyridine-*d*_5_, 600 MHz) *δ*: 1.82 and 1.18 (2H, m, H_2_-1), 1.92 and 1.85 (2H, m, H_2_-2), 4.14 (1H, dd, *J* = 10.8, 4.9 Hz, H-3β), 1.90 (1H, overlap, H-5), 2.05–2.00 (2H, overlap, H_2_-6), 5.66 (1H, d, *J* = 2.1 Hz, H-7), 2.40 (1H, overlap, H-9β), 1,75 and 1.20 (2H, m, H_2_-11), 2.20 and 1.65 (2H, m, H_2_-12), 2.50 (1H, br. d, *J* = 12.4 Hz, H-13α), 4.58 (1H, br. s, H-14α), 4.56 and 4.65 (2H, 2d, *J* = 14.0 Hz, H_2_-16), 5.57 and 5.28 (2H, br. s, H_2_-17), 4.10 and 3.59 (2H, d, *J* = 10.8 Hz, H_2_-18), 1.15 (Me, s, H_3_-19), 0.92 (Me, s, H_3_-20); ^13^C-NMR (pyridine-*d*_5_, 125 MHz) *δ*: 38.1 (t, C-1), 24.3 (t, C-2), 77.3 (d, C-3), 36.6 (s, C-4), 45.5 (d, C-5), 22.4 (t, C-6), 123.2 (d, C-7), 141.5 (s, C-8), 48.5 (d, C-9), 35.1 (s, C-10), 22.4 (t, C-11), 25.3 (t, C-12), 47.2 (d, C-13), 74.4 (d, C-14), 152.5 (s, C-15), 64.6 (t, C-16), 110.6 (t, C-17), 72.2 (t, C-18), 12.9 (q, C-19), 15.8 (q, C-20).

Compound **7**: White amorphous powder; UV (MeOH), *λ*_max_ 206 nm; IR (KBr), *ν*_max_ 3386, 2930, 1630, 1444, 1383, 1051, 1033 cm^−1^; ^1^H-NMR (pyridine-*d*_5_, 400 MHz) *δ*: 1.76 and 1.18 (each 1H, m, H_2_-1), 1.78 (2H, m, H_2_-2), 4.26 (1H, overlap, H-3), 1.71 (1H, overlap, H-5), 2.52 (1H, br d, *J* = 12.5 Hz, H-6a), 1.87 (1H, overlap, H-6b), 5.33 (1H, br s, H-7), 1.68 (1H, overlap, H-9), 1.88 (2H, m, H_2_-11), 1.86 and 1.12 (each 1H, overlap, H_2_-12), 1.79 (1H, m, H-13), 1.68 (1H, overlap, H-14a), 1.09 (1H, m, H-14b), 1.70 (1H, overlap, H-15), 4.24–4.15 (4H, m, H_2_-16 and H_2_-17), 4.12 and 3.65 (each 1H, d, *J* = 9.5 Hz, H_2_-18), 1.13 (Me, s, H_3_-19), 0.88 (Me, s, H_3_-20). ^13^C-NMR (pyridine-*d*_5_, 100 MHz) *δ*: 38.1 (t, C-1), 27.9 (t, C-2), 73.5 (d, C-3), 43.1 (s, C-4), 43.0 (d, C-5), 39.7 (t, C-6), 120.3 (d, C-7), 137.7 (s, C-8), 52.9 (d, C-9), 35.3 (s, C-10), 23.4 (t, C-11), 30.2 (t, C-12), 37.0 (d, C-13), 25.9 (t, C-14), 49.2 (d, C-15), 62.1 (t, C-16), 62.0 (t, C-17), 67.5 (t, C-18), 13.2 (q, C-19), 15.9 (t, C-20).

Compound **8**: White amorphous powder; UV (MeOH), λ_max_ 202 nm; IR (KBr), ν_max_ 3423, 2937, 2866, 1720, 1636 cm^−1^; ^1^H-NMR (pyridine-*d*_5_, 400 MHz) *δ*: 1.81 and 1.19 (each 1H, overlap, H_2_-1), 1.81–1.92 (2H, m, H_2_-2), 4.00 (1H, dd, *J* = 4.5, 10.6 Hz, H-3β), 1.65 (1H, dd, *J* = 3.9, 11.9 Hz, H-5β), 1.78 and 1.15 (each 1H, overlap, H_2_-6), 5.50 (1H, br d, *J* = 4.5 Hz, H-7), 2.39 (1H, br d, *J* = 11.5 Hz, H-9β), 1.49 and 2.00 (each 1H, overlap, H_2_-11), 2.29 and 1.70 (each 1H, m, H_2_-12), 2.48 (1H, br d, *J* = 12.5 Hz, H-13*α*), 4.59 (1H, br s, H-14α), 5.57 and 5.29 (each 1H, br s, H2-16), 4.66 and 4.52 (each 1H, d, *J* = 12.0 Hz, H-17a), 9.44 (1H, s, H-18), 1.42 (3H, s, Me-19), 0.83 (3H, s, Me-20), 6.53 (1H, br s, OH-3), 5.89 (1H, br s, OH-14), 6.57 (1H, br s, OH-17); ^13^C-NMR (pyridine- d_5_, 125 MHz) *δ*: 37.8 (t, C-1), 27.3 (t, C-2), 72.4 (d, C-3), 55.7 (s, C-4), 41.9 (d, C-5), 25.6 (t, C-6), 123.0 (d, C-7), 141.7 (s, C-8), 48.2 (d, C-9), 34.1 (s, C-10), 24.6 (t, C-11), 23.8 (t, C-12), 47.2 (d, C-13), 74.4 (d, C-14), 152.6 (s, C-15), 64.8 (t, C-16), 110.7 (t, C-17), 206.8 (d, C-18), 9.8 (q, C-19), 15.7 (q, C-20).

### 3.4. Cytotoxicity Assays 

The human tumor cell lines HL-60, SMMC-7721, A-549, MCF-7, and SW-480 were used, which were obtained from ATCC (Manassas, VA, USA). All cells were cultured in RPMI-1640 or DMEM medium (Hyclone, Logan, UT, USA), supplemented with 10% fetal bovine serum (Hyclone) at 37 °C in a humidified atmosphere with 5% CO_2_. Cell viability was assessed by conducting colorimetric measurements of the amount of insoluble formazan formed in living cells based on the reduction of MTS (Sigma, St. Louis, MO, USA) [25]. Briefly, adherent cells (100 μL) were seeded into each well of a 96-well cell culture plate and were allowed to adhere for 12 h before test compound addition, while suspended cells were seeded just before test compound addition, both with an initial density of 1 × 10^5^ cells/mL in 100 μL of 20% SDS-50% DMF after removal of 100 μL of medium. The optical density of the lysate was measured at 595 nm in a 96-well microtiter plate reader (Bio-Rad 680). The IC_50_ value of each compound was calculated by Reed and Muench’s method [26].

### 3.5. Nitric Oxide Production in RAW264.7 Macrophages 

Murine monocytic RAW264.7 macrophages were dispensed into 96-well plates (2 × 10^5^ cells/well) containing RPMI 1640 medium (Hyclone) with 10% FBS under a humidified atmosphere of 5% CO_2_ at 37 °C. After 24 h pre-incubation, cells were treated with serial dilutions in the presence of 1 μg/mL LPS for 18 h. Each compound was dissolved in DMSO and further diluted in medium to produce different concentrations. NO production in each well was assessed by adding 100 μL of Griess reagent (reagent A and reagent B, respectively, Sigma) to 100 μL of each supernatant from LPS (Sigma)-treated or LPS- and compound-treated cells in triplicate. After 5 min of incubation, the absorbance was measured at 570 nm with a 2104 Envision multitable plate reader (Perkin-Elmer Life Sciences, Inc., Boston, MA, USA). MG-132 was used as a positive control [27].

### 3.6. Determination of Cytotoxic Effects

The cytotoxicity of the tested compounds was evaluated using an MTS assay. Briefly, RAW264.7 cells, 2 × 10^5^ cells/well, were seeded in 96-well plates. After 24 h incubation, cells were treated with or without test compounds at given concentrations for 18 h. Then, MTS was added to each well and the plates were kept for 4 h. The testing compounds were dissolved in DMSO, and the absorbance was read at 490 nm. Cytotoxicity was calculated by the cell viability of the cells without compounds as 100%.

## 4. Conclusions

Four new *ent*-abietane diterpenoids, together with four known ones, were isolated from *Isodon serra*, collected in the E′mei Mountain of China, all of which were *ent*-abietane type discovered for the first time from this species. In vitro bioactive tests revealed that compound **1** exhibited some cytotoxicity against five human cell lines (HL-60, SMMC-7721, A-549, MCF-7, SW480), and showed significant inhibitory activity towards NO production with an IC_50_ value of 1.8 μM.

## Figures and Tables

**Table 1:** ^1^H-NMR spectroscopic data for compounds **1**–**4** in pyridine-*d*_5_ (*δ* in ppm, *J* in Hz).

Position	1 ^a^	2 ^a^	3 ^b^	4 ^c^
1a	4.81 (dd, 11.5, 5.1)	1.80 (dt, 13.2, 3.2)	1.67 (dt, 13.1, 3.3)	1.60 (dt, 13.1, 3.3)
1b		1.19 (m)	1.32 (dt, 13.1, 3.3)	1.21 (dt, 13.1, 3.3)
2a	1.80 (m)	1.90 (2H, overlap)	1.95 (overlap)	1.92 (overlap)
2b	1.55 (overlap)		1.88 (overlap)	1.85 (m)
3a	1.32 (dt, 13.5, 3.3)	4.12 (dd, 11.0, 4.6)	4.18 (br d, 11.1)	4.12 (br d, 8.8)
3b	1.17 (overlap)			
5	1.41 (dd, 11.4, 5.8)	1.70 (overlap)	2.61 (overlap)	2.27 (dd, 13.2, 2.5)
6a	2.03 (2H, overlap)	2.01 (overlap)	1.78 (dd, 13.6, 2.8)	1.68 (overlap)
6b		1.48 (m)	1.57 (dt, 13.6, 2.8)	1.44 (dt, 13.2, 2.8)
7		5.26 (d, 4.1)	4.40 (overlap)	3.47 (t, 2.8)
8	2.16 (t, 11.0)			
9	1.92 (m)	1.70 (overlap)	2.64 (overlap)	2.11 (br s)
11a	1.62 (m)	1.70 (overlap)	1.74 (overlap)	1.65 (overlap)
11b	1.49 (m)	1.05 (m)	1.37 (overlap)	1.30 (overlap)
12a	1.19 (overlap)	1.25 (m)	1.92 (overlap)	1.92 (overlap)
12b	2.06 (overlap)	2.03 (overlap)	1.37 (overlap)	1.30 (overlap)
13	3.30 (m)	1.85 (m)	2.89 (br s)	2.88 (br s)
14a	5.31 (t, 9.8)	2.57 (br d, 13.9)	5.86 (s)	5.73 (s)
14b		2.04 (overlap)		
15		1.93 (overlap)		
16		4.19 (2H, overlap)	4.42 (2H, overlap)	4.47 (2H, s)
17a	6.33 (d, 3.1)	4.23 (2H, overlap)	5.44 (br s)	5.54 (br s)
17b	5.52 (d, 3.1)		4.98 (br s)	5.10 (br s)
18 18b	0.74 (3H, s)	9.62 (s)	9.69 (s) 3.54 (2H, d, 10.6)	9.61 (s)
19	1.07 (3H, s)	1.45 (3H, s)	1.42 (3H, s)	1.37 (3H, s) 3.54 (2H, d, 10.6)
20a	4.35 (d, 10.3)	0.82 (3H, s)	0.85 (3H, s)	0.80 (3H, s)
20b	4.21 (d, 10.3)			
OAc-1	2.07 (s)			
OMe				3.16 (3H, s)

^a^ Recorded at 600 MHz, ^b^ Recorded at 400 MHz, ^c^ Recorded at 500 MHz in pyridine-*d*_5_. The assignments were based on the distortionless enhancement by polarization transfer (DEPT), and heteronuclear single quantum correlation (HSQC), ^1^H-^1^H COSY, and HMBC experiments.

**Table 2:** ^13^C-NMR spectroscopic data for compounds **1**–**4** in pyridine-*d*_5_ (*δ* in ppm).

Position	1 ^a^	2 ^a^	3 ^c^	4 ^c^
1	76.5 (d)	38.2 (t)	37.1 (t)	36.8 (t)
2	25.8 (t)	27.8 (t)	27.5 (t)	27.4 (t)
3	38.6 (t)	72.8 (d)	71.8 (d)	71.7 (d)
4	34.1 (s)	56.2 (s)	56.2 (s)	56.0 (s)
5	49.8 (d)	42.6 (d)	39.1 (d)	39.6 (d)
6	36.1 (t)	25.2 (t)	32.4 (t)	30.7 (t)
7	96.8 (s)	119.7 (d)	71.9 (d)	81.3 (d)
8	45.4 (d)	138.6 (s)	141.3 (s)	135.8 (s)
9	47.0 (d)	52.9 (d)	46.5 (d)	46.5 (d)
10	37.7 (s)	34.8 (s)	37.7 (s)	37.3 (s)
11	21.9 (t)	26.3 (t)	22.5 (t)	22.4 (t)
12	27.7 (t)	30.5 (t)	29.5 (t)	29.8 (t)
13	39.5 (d)	37.4 (d)	39.4 (d)	39.9 (d)
14	77.7 (d)	40.1 (t)	129.3 (d)	132.6 (d)
15	140.9 (s)	49.6(d)	155.0 (s)	155.2 (s)
16	170.8 (s)	62.4 (t)	64.3 (t)	64.3 (t)
17	121.8 (t)	62.3 (t)	107.9 (t)	108.3 (t)
18	31.8 (q)	207.3 (d)	206.8 (d)	206.7 (d)
19	20.3 (q)	10.3 (q)	9.7 (q)	9.6 (q)
20	64.1 (t)	16.0 (q)	14.4 (q)	14.5 (q)
OAc-1	170.6 (s)			
	21.9 (q)			
OMe				54.8 (q)

^a^ Recorded at 150 MHz, ^b^ Recorded at 100 MHz, ^c^ Recorded at 125 MHz. The assignments were based on the DEPT, HSQC, ^1^H-^1^H COSY, and HMBC experiments.

## Supplementary Materials

Supplementary materials are available online.
