# Supplementary material for: Ent-Abietanoids Isolated from Isodon serra
Source: Molecules. 2017 Feb 17;22(2):309. doi: 10.3390/molecules22020309 (PMC6155895; doi:10.3390/molecules22020309)
Supplement: Supplementary file 1 [file molecules-22-00309-s001.pdf]

Supplementary Materials: *ent*-Abietanoids Isolated from *Isodon serra*

Jun Wan[1,2], **Hua-Yi Jiang**[1,2], **Jian-Wei Tang**[1,2], **Xing-Ren Li**[1,2], **Xue Du**[1], **Yan Li**[1], **Han-Dong Sun**[1] and **Jian-Xin Pu**[1,*]

[1] State Key Laboratory of Phytochemistry and Plant Resources in West China, Kunming Institute of Botany, Chinese Academy of Sciences, Kunming 650201, P.R. China; pujianxin@mail.kib.ac.cn
[2] University of Chinese Academy of Sciences, Beijing 100039, P.R. China; wanjun@imm.ac.cn

Corresponding author: Jian-Xin Pu

State Key Laboratory of Phytochemistry and Plant Resources in West China, Kunming Institute of Botany, Chinese Academy of Sciences, Kunming 650201, PR China

Tel.: +86-871-65223616.

E-mail address: pujianxin@mail.kib.ac.cn (J.-X. Pu).

Contents of Supporting Information

No.	Contents	Page
1.	Figure S1. ^{1}H (600 MHz) and ^{13}C NMR (150 MHz)spectra of serrin K (**1**) in C_5D_5N	3
2.	Figure S2. HSQC and ^{1}H-^{1}H COSY spectra of serrin K (**1**) in C_5D_5N (600 MHz)	4
3.	Figure S3. HMBC and ROESY spectra of serrin K (**1**) in C_5D_5N (600 MHz)	5
4.	Figure S4. HR-ESI-MS and ORD spectra of serrin K (**1**)	6
5.	Figure S5. UV (in MeOH) and IR (KBr) spectra of serrin K (**1**)	7
6.	Figure S6. ^{1}H (600 MHz) and ^{13}C NMR (150 MHz) spectra of xerophilusin XVII (**2**) in C_5D_5N	8
7.	Figure S7. HSQC and ^{1}H-^{1}H COSY spectra of xerophilusin XVII (**2**) in C_5D_5N (600 MHz)	9
8.	Figure S8. HMBC and ROESY spectra of xerophilusin XVII (**2**) in C_5D_5N (600 MHz)	10
9.	Figure S9. HR-ESI-MS spectra of xerophilusin XVII (**2**)	11
10.	Figure S10. UV (in MeOH) and IR (KBr) spectra of xerophilusin XVII (**2**)	12
11.	Figure S11. ^{1}H (400 MHz) and ^{13}C NMR (125 MHz) spectra of enanderianin Q (**3**) in C_5D_5N	13
12.	Figure S12. HSQC and ^{1}H-^{1}H COSY spectra of enanderianin Q (**3**) in C_5D_5N (500 MHz)	14
13.	Figure S13. HMBC and ROESY spectra of enanderianin Q (**3**) in C_5D_5N (500 MHz)	15
14.	Figure S14. HR-ESI-MS and ORD spectra of enanderianin Q (**3**)	16
15.	Figure S15. UV (in MeOH) and IR (KBr) spectra of enanderianin Q (**3**)	17
16.	Figure S16. ^{1}H (500 MHz) and ^{13}C NMR (125 MHz) spectra of enanderianin R (**4**) in C_5D_5N	18
17.	Figure S17. HSQC and ^{1}H-^{1}H COSY spectra of enanderianin R (**4**) in C_5D_5N (500 MHz)	19
18.	Figure S18. HMBC and ROESY spectra of enanderianin R (**4**) in C_5D_5N (500 MHz)	20
19.	Figure S19. HR-ESI-MS and ORD spectra of enanderianin R (**4**)	21
20.	Figure S20. UV (in MeOH) and IR (KBr) spectra of enanderianin R (**4**)	22

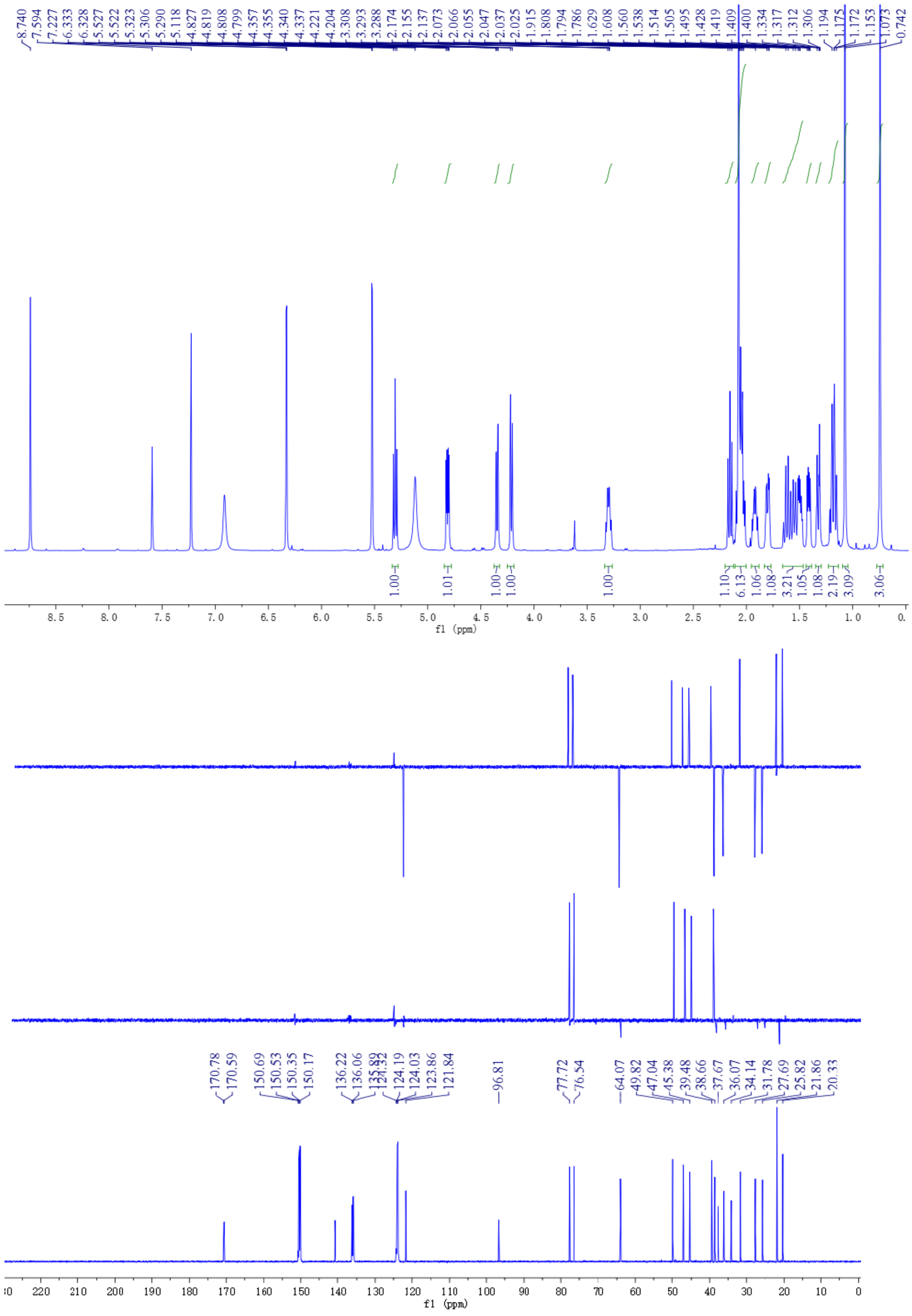

Figure S1. ^{1}H (600 MHz) and ^{13}C NMR (150 MHz) spectra of serrin K (**1**) in C_5D_5N.

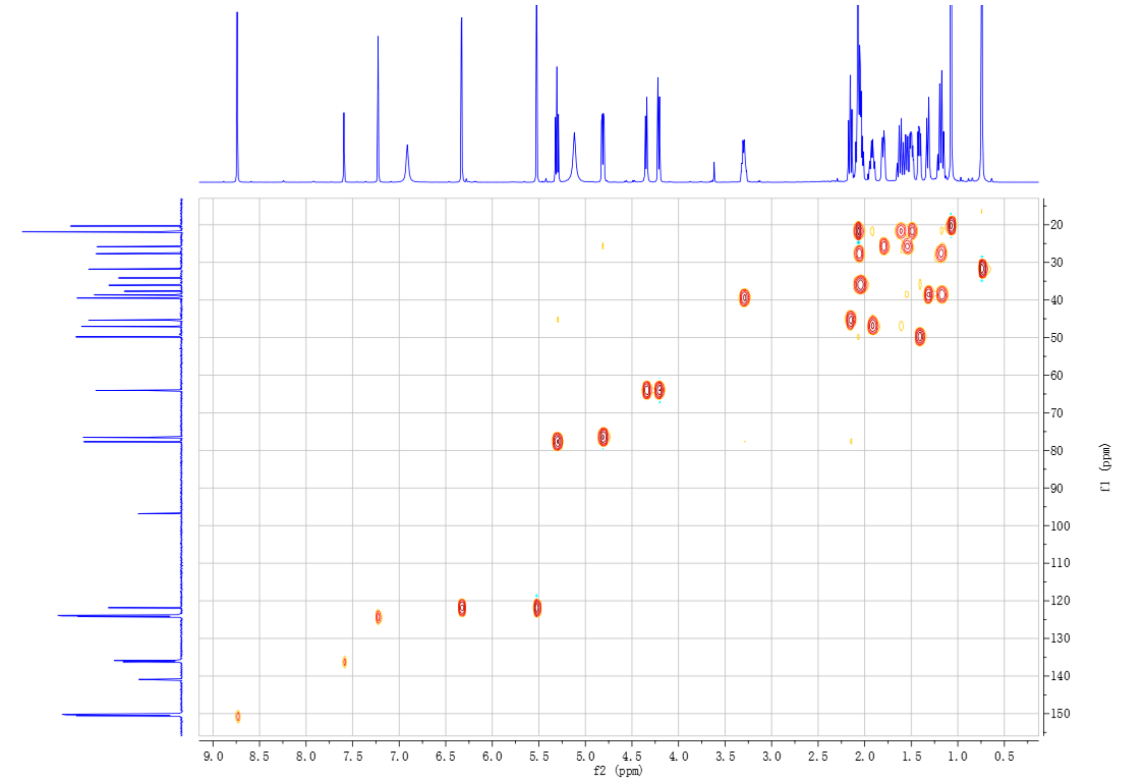

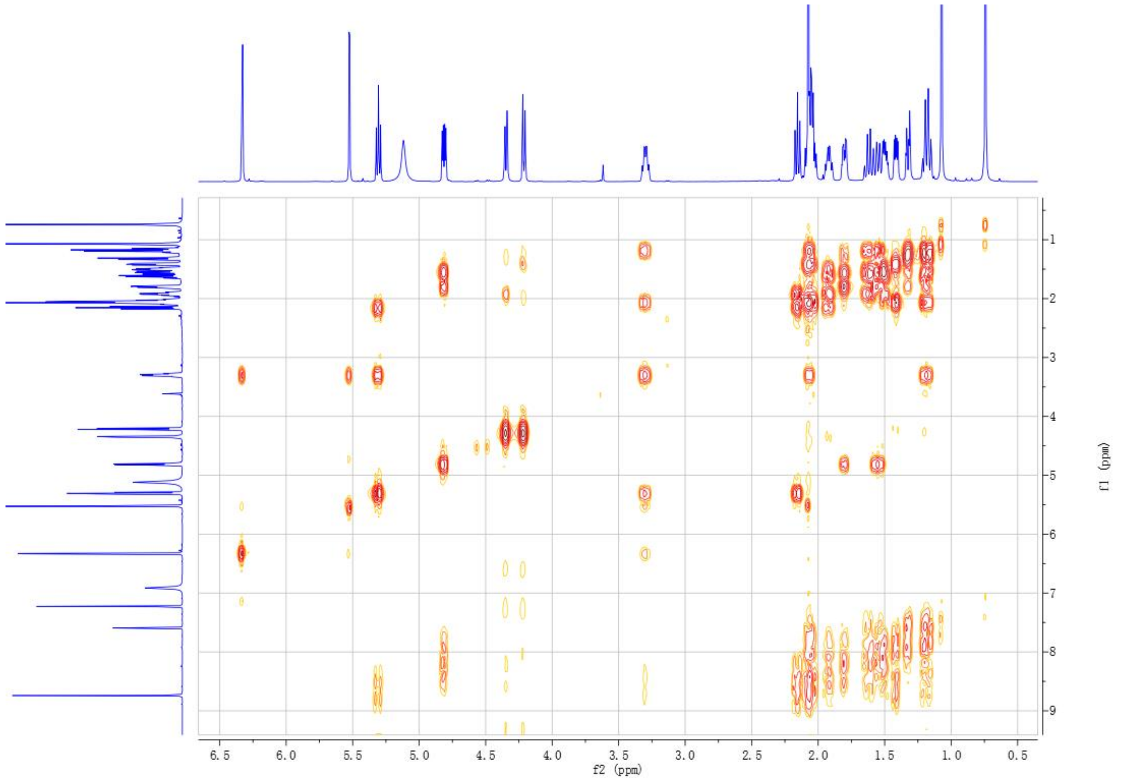

Figure S2. HSQC and ^{1}H-^{1}H COSY spectra of serrin K (**1**) in C_5D_5N (600 MHz).

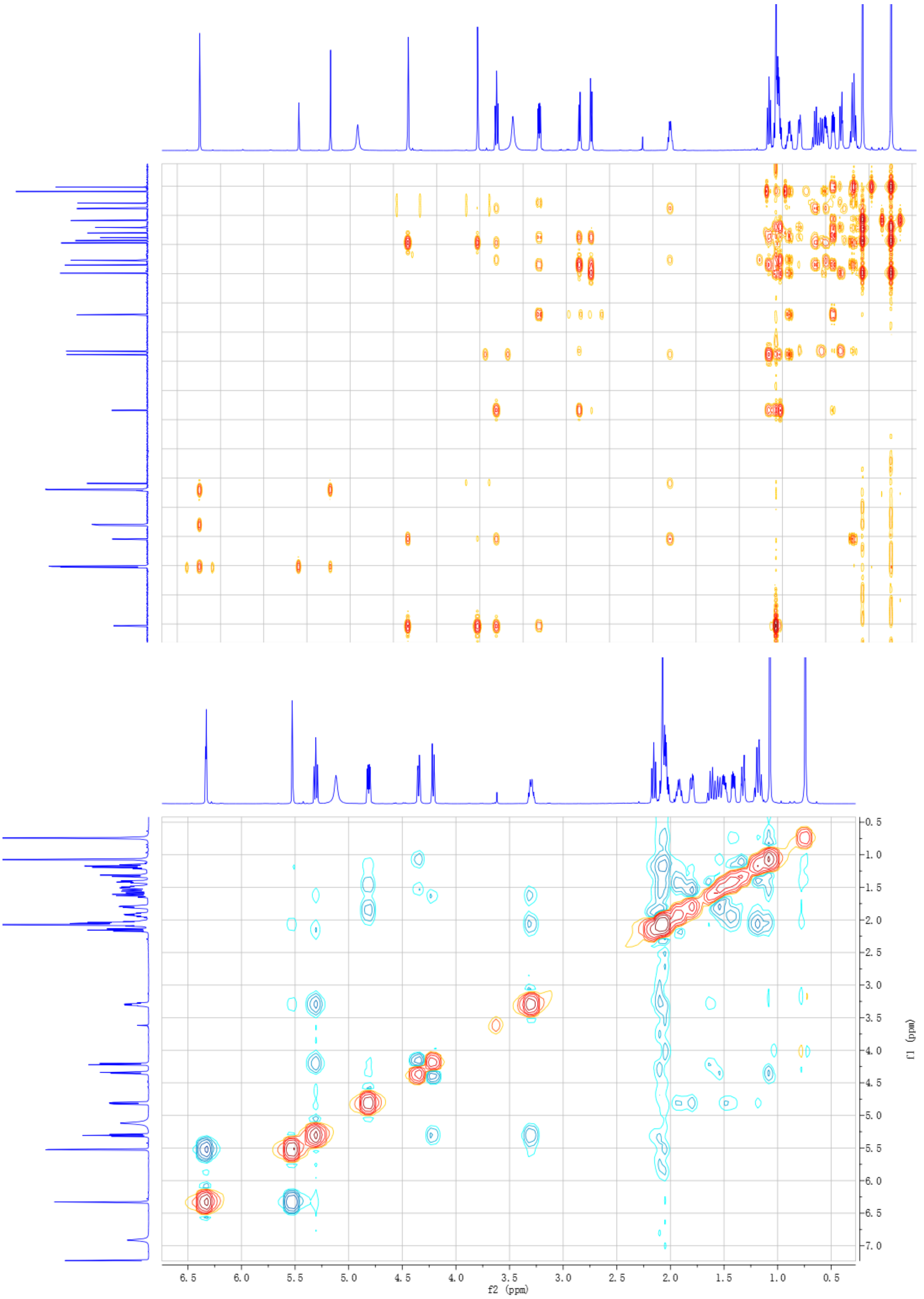

Figure S3. HMBC and ROESY spectra of serrin K (**1**) in C_5D_5N (600 MHz).

Qualitative Analysis Report

**Data Filename**	swj45.d	**Sample Name**	swj45
**Sample Type**	Sample	**Position**	P1-F3
**Instrument Name**	Instrument 1	**User Name**	
**Acq Method**	SIBU.m	**Acquired Time**	4/27/2015 4:15:07 PM
**IRM Calibration Status**	Success	**DA Method**	Default.m
**Comment**			

Sample Group **Info.**

Acquisition SW Version 6200 series TOF/6500 series Q-TOF B.05.01 (B5125.2)

User Spectra

Fragmentor Voltage	Collision Energy	Ionization Mode
135	0	ESI

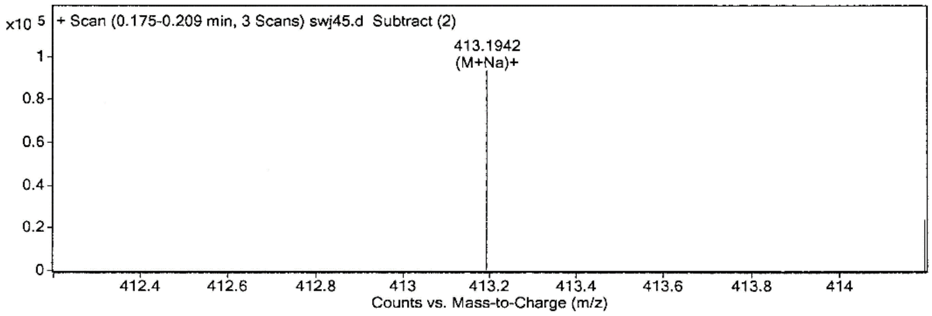

Peak List

m/z	z	Abund	Formula	Ion
277.1286	1	81398.48		
299.1109	1	363410.34		
315.0845	1	160241.05		
366.2465	1	80564.24		
408.2385	1	92039.3		
413.1942	1	93982.81	C22 H30 O6	(M+Na)+

Formula Calculator Element Limits

Element	Min	Max
C	3	60
H	0	120
O	0	30

Formula Calculator Results

Formula	CalculatedMass	CalculatedMz	Mz	Diff. (mDa)	Diff. (ppm)	DBE
C22 H30 O6	390.2042	413.1935	413.1942	-0.8	-1.9	8.0000

Optical rotation measurement

Model : P-1020 (A060460638)

No.	Sample	Mode	Data	Monitor<br>Blank	Temp.<br>Cell<br>Temp Point	Date<br>Comment<br>Sample Name	Light<br>Filter<br>Operator	Cycle Time<br>Integ Time
No.1	8 (1/3)	Sp.Rot	-48.7120	-0.0397<br>0.0000	22.9<br>50.00<br>Cell	Fri Apr 24 18:15:01 2015<br>0.00163g/mL MeOH<br>SWJ45	Na<br>589nm	2 sec<br>10 sec
No.2	8 (2/3)	Sp.Rot	-48.2210	-0.0393<br>0.0000	23.0<br>50.00<br>Cell	Fri Apr 24 18:15:15 2015<br>0.00163g/mL MeOH<br>SWJ45	Na<br>589nm	2 sec<br>10 sec
No.3	8 (3/3)	Sp.Rot	-48.4660	-0.0395<br>0.0000	23.0<br>50.00<br>Cell	Fri Apr 24 18:15:28 2015<br>0.00163g/mL MeOH<br>SWJ45	Na<br>589nm	2 sec<br>10 sec

-48.4663°

Figure S4. HR-ESI-MS and ORD spectra of serrin K (**1**).

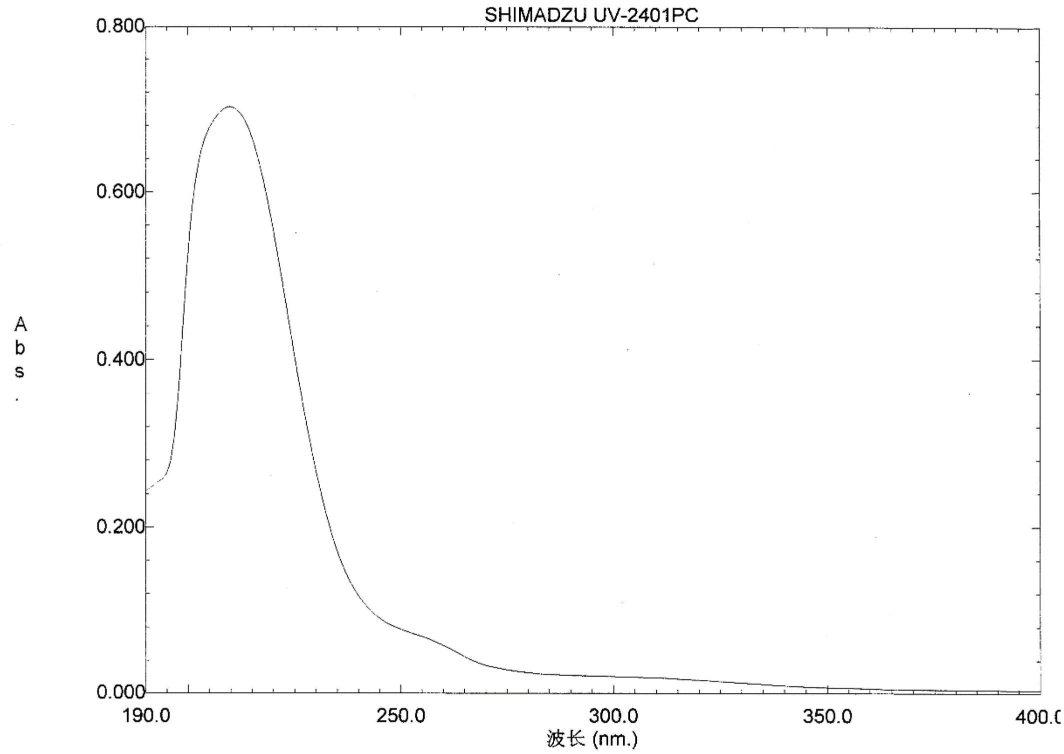

文件名: SWJ45

SWJ45 ———

样品浓度：0.0326毫克/毫升
溶剂：甲醇

创建于: 10:22 15-04-27
数据: 原始

测量模式: Abs.
扫描速度: 中速
狭缝: 5.0
采样间隔: 0.2

否.	波长 (nm.)	Abs.
1	209.80	0.7024

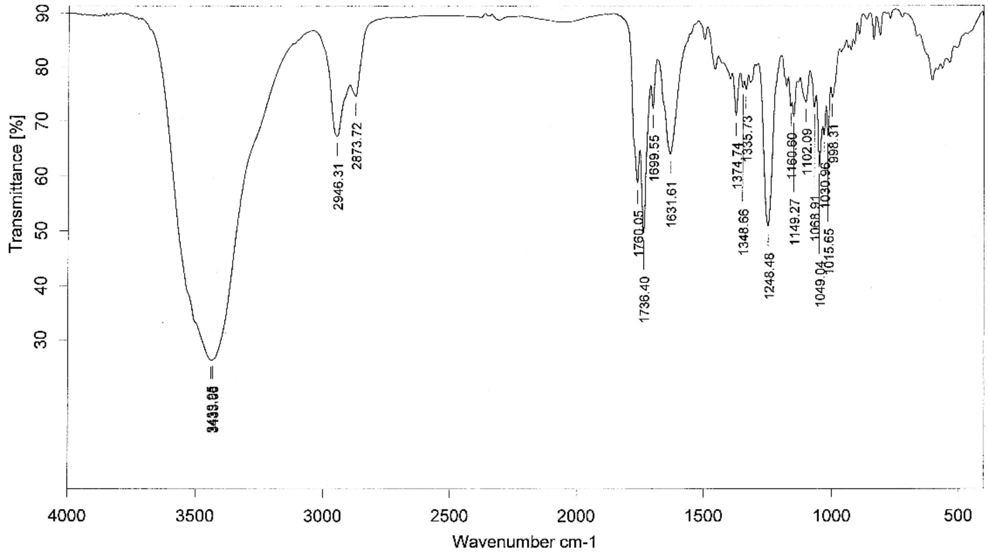

Sample : swj45	Frequency Range : 399.246 - 3996.32	Measured on : 29/04/2015	
Technique : KBr压片	Resolution : 4	Instrument : Tensor27	Sample Scans : 16
Customer : 150429IR3	Zerofilling : 2	Acquisition : Double Sided,For	

Figure S5. UV (in MeOH) and IR (KBr) spectra of serrin K (**1**).

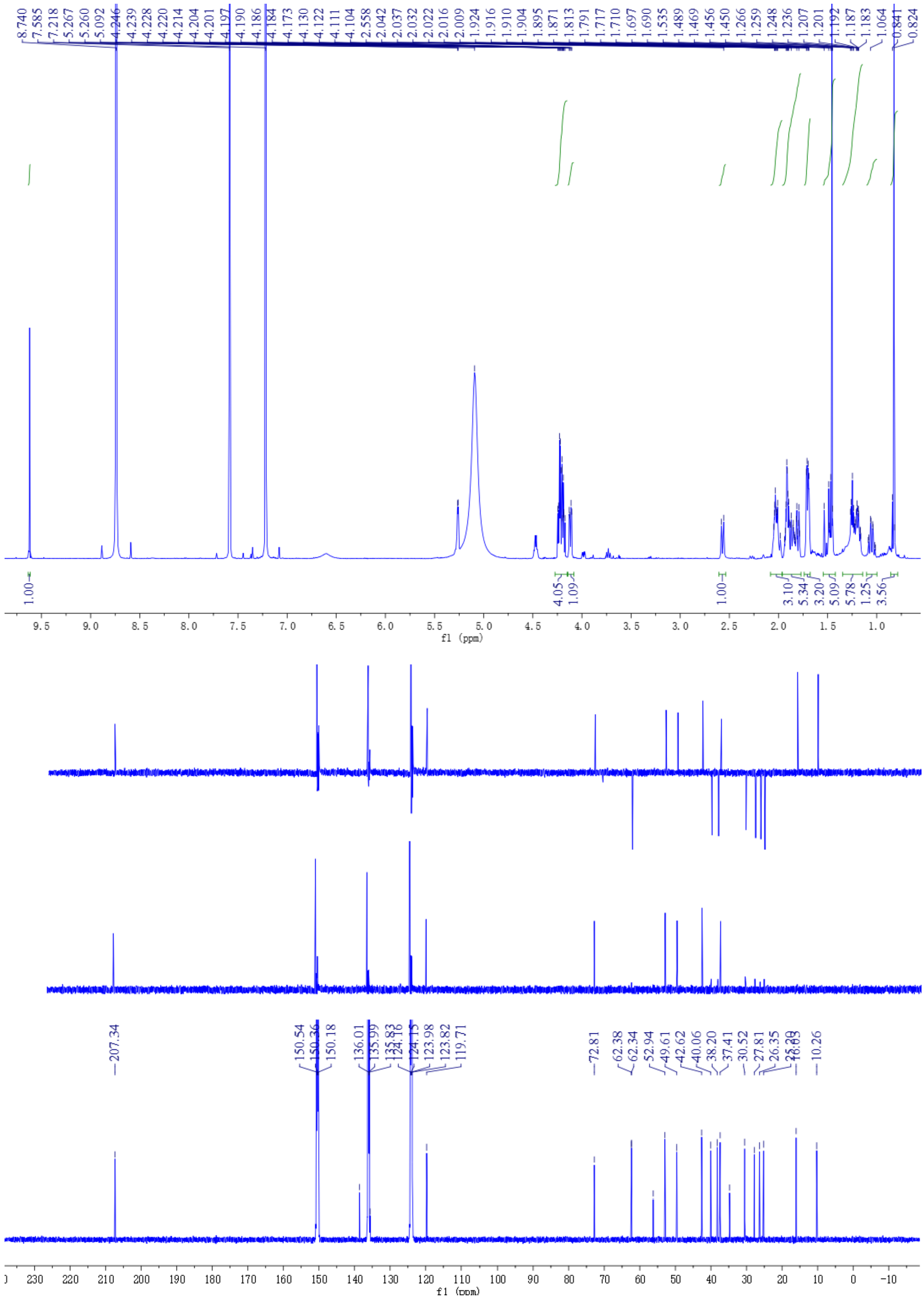

Figure S6. ^{1}H (600 MHz) and ^{13}C NMR (150 MHz) spectra of xerophilusin XVII (**2**) in C_5D_5N.

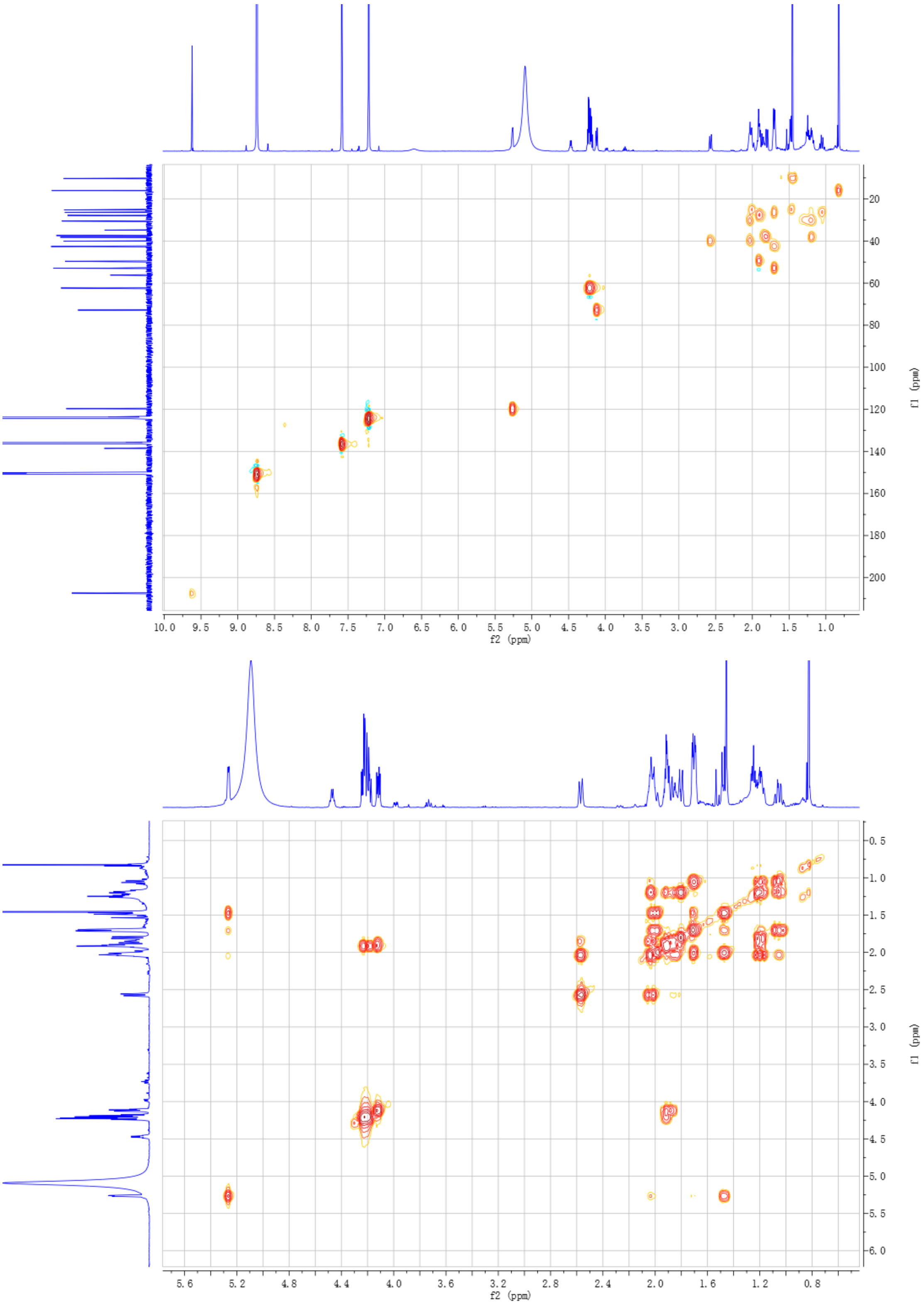

Figure S7. HSQC and ^{1}H-^{1}H COSY spectra of xerophilusin XVII (**2**) in C_5D_5N (600 MHz).

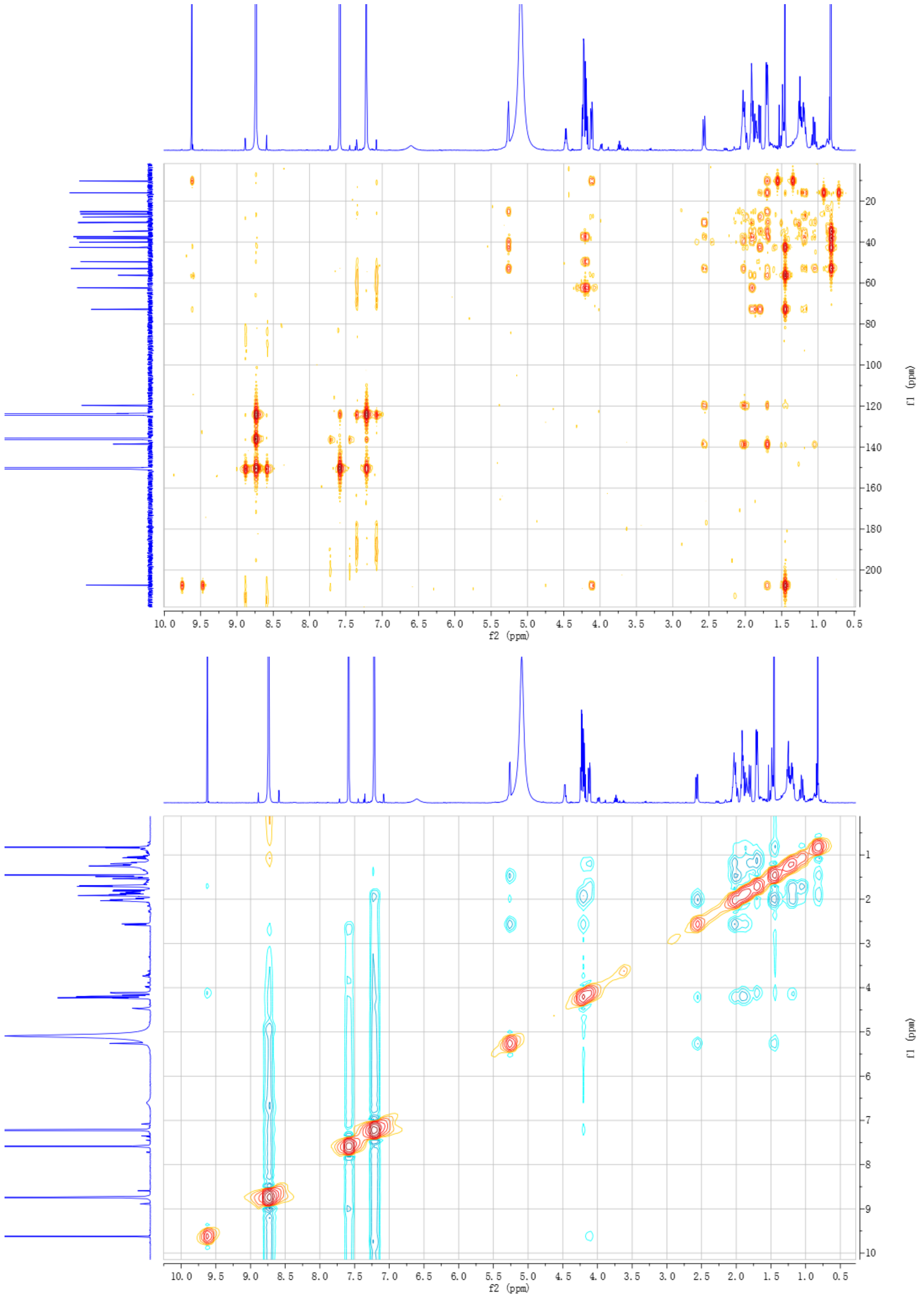

Figure S8. HMBC and ROESY spectra of xerophilusin XVII (**2**) in C_5D_5N (600 MHz).

Qualitative Analysis Report

**Data Filename**	SWJ29.d	**Sample Name**	SWJ29
**Sample Type**	Sample	**Position**	P1-C6
**Instrument Name**	Instrument 1	**User Name**	
**Acq Method**	SIBU.m	**Acquired Time**	4/30/2015 3:20:16 PM
**IRM Calibration Status**	Success	**DA Method**	Default.m
**Comment**			

Sample Group **Info.**

Acquisition SW 6200 series TOF/6500 series

Version Q-TOF B.05.01 (B5125.2)

User Spectra

Fragmentor Voltage	Collision Energy	Ionization Mode
135	0	ESI

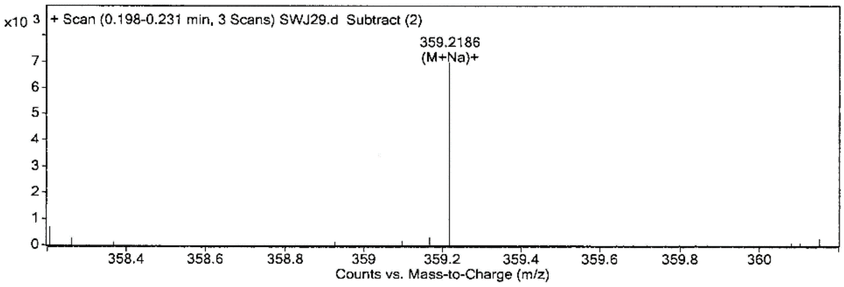

Peak List

m/z	z	Abund
197.1169	1	13136.37
391.2113	1	20579.63
594.2544	1	47332.64
595.2576	1	15770.32
599.2083	1	21739.52
615.1832	1	21020.84

Formula Calculator Element Limits

Element	Min	Max
C	3	60
H	0	120
O	0	30

Formula Calculator Results

Formula	CalculatedMass	CalculatedMz	Mz	Diff. (mDa)	Diff. (ppm)	DBE
C20 H32 O4	336.2301	359.2193	359.2186	0.5	1.6	5.0000

Figure S9. HR-ESI-MS spectra of xerophilusin XVII (**2**).

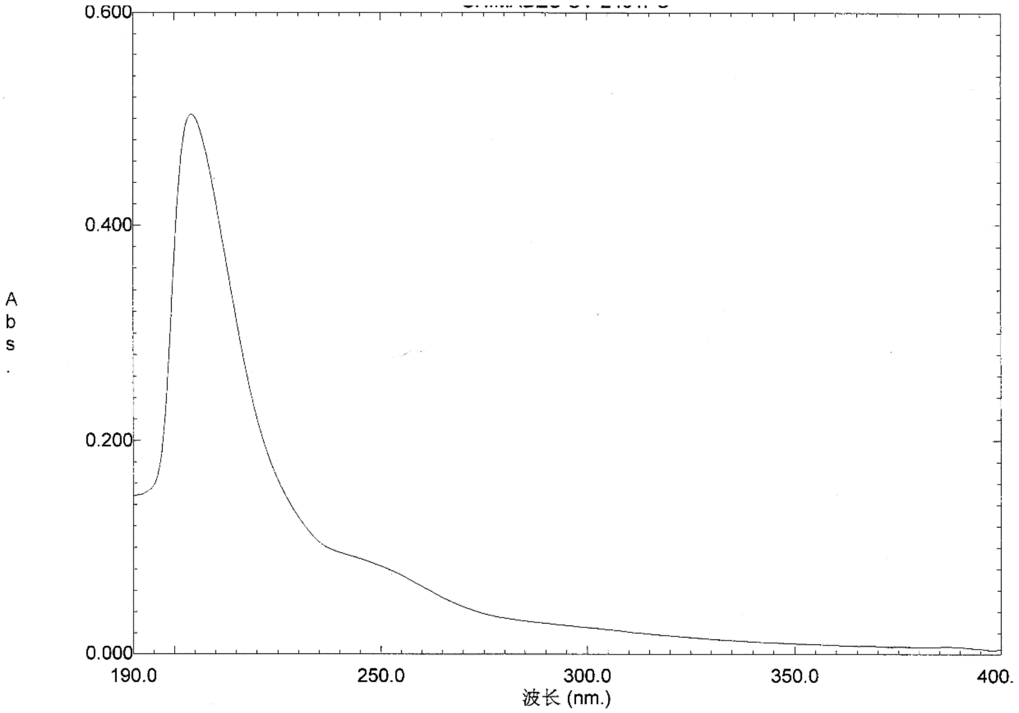

文件名: SWJ29

SWJ29 ———

样品浓度：0.0306毫克/毫升
溶剂：甲醇

创建于: 19:03 15-05-07
数据: 原始

测量模式: Abs.
扫描速度: 中速
狭缝: 5.0
采样间隔: 0.2

否.	波长 (nm.)	Abs.
1	204.20	0.5040
2	241.40	0.0937

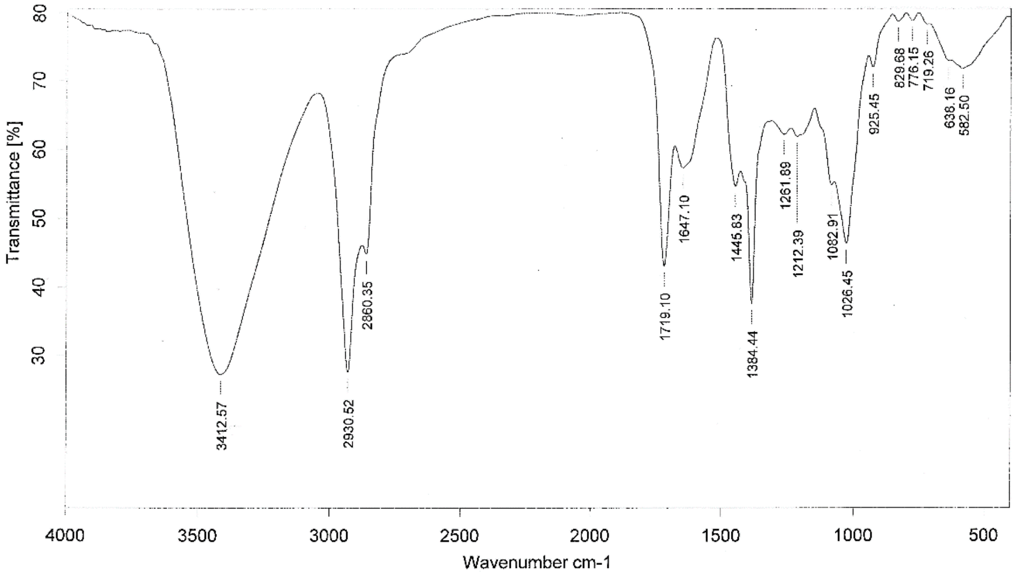

Sample : swj29	Frequency Range : 399.246 - 3996.32	Measured on : 11/07/2016	
Technique : KBr压片	Resolution : 4	Instrument : Tensor27	Sample Scans : 16
Customer : 160711IR1	Zerofilling : 2	Acquisition : Double Sided,For	

Figure S10. UV (in MeOH) and IR (KBr) spectra of xerophilusin XVII (**2**).

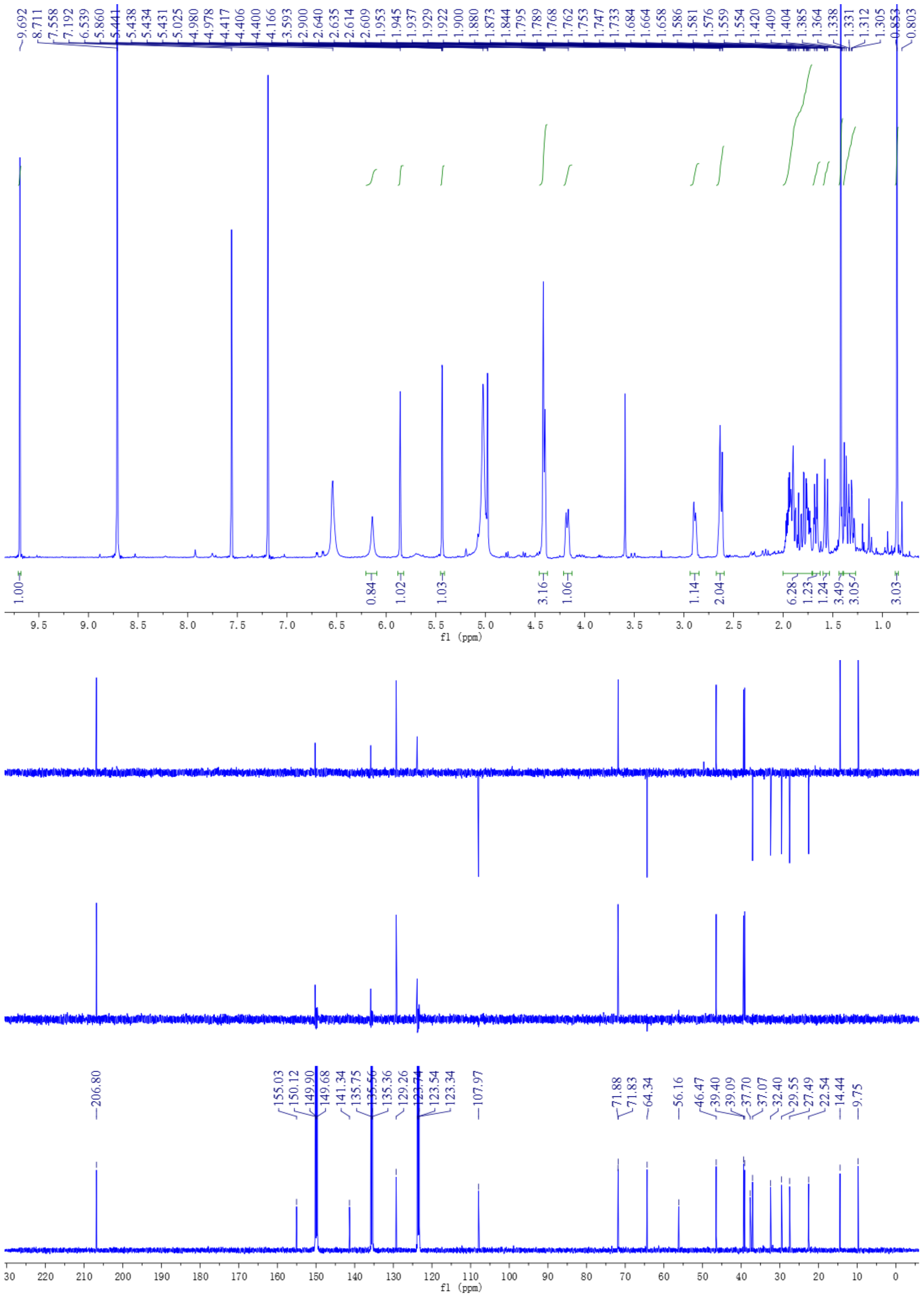

Figure S11. ^{1}H (400 MHz) and ^{13}C NMR (125 MHz) spectra of enanderianin Q (**3**) in C_5D_5N.

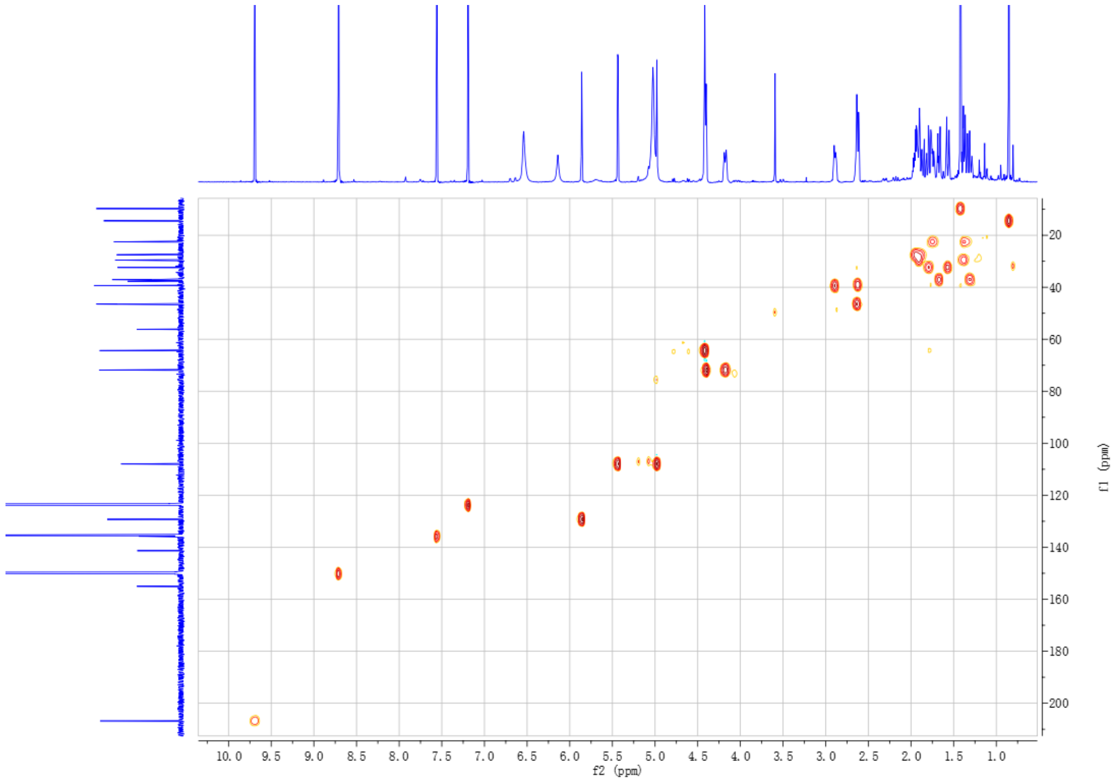

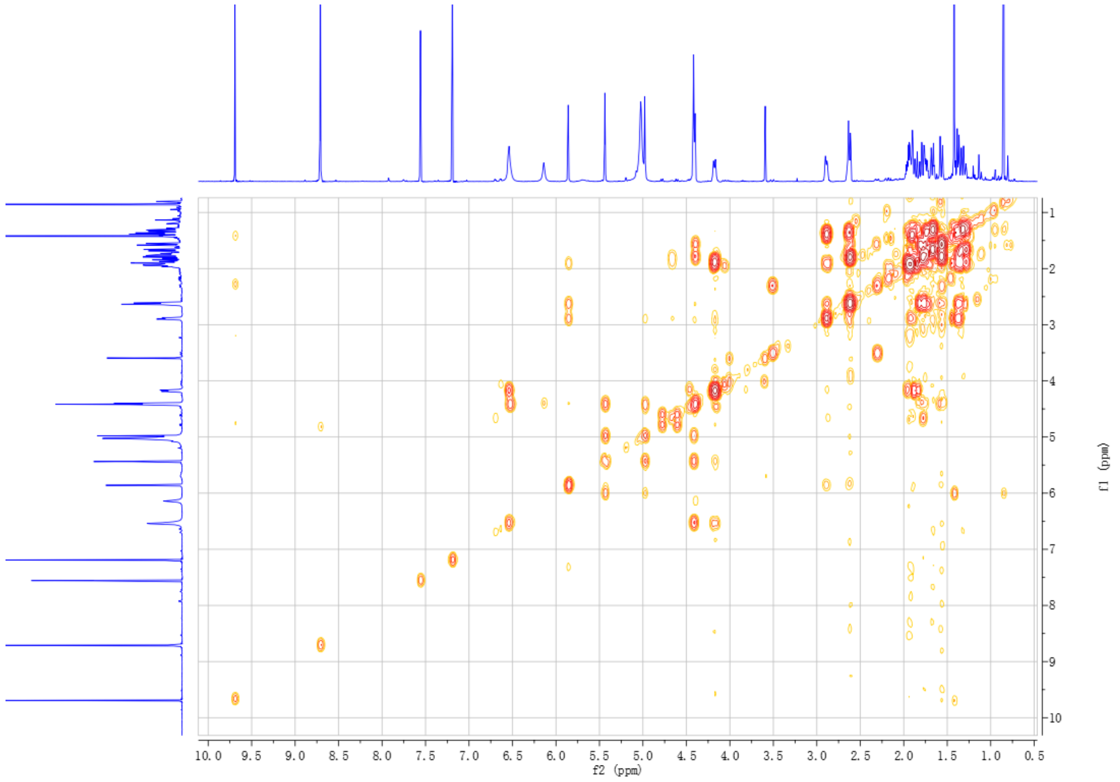

Figure S12. HSQC and ^{1}H-^{1}H COSY spectra of enanderianins Q (**3**) in C_5D_5N (500 MHz).

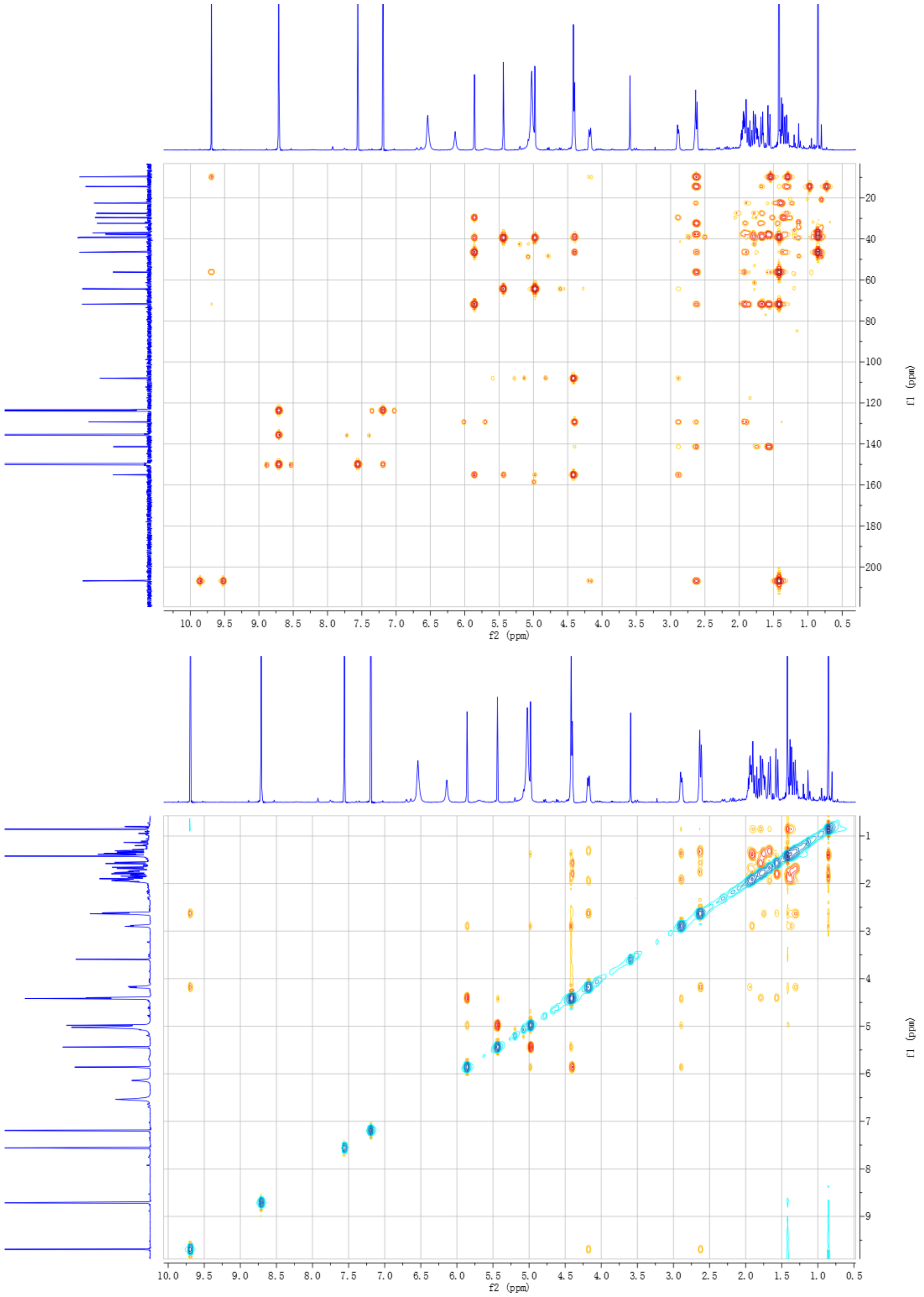

Figure S13. HMBC and ROESY spectra of enanderianin Q (**3**) in C_5D_5N (500 MHz).

Qualitative Analysis Report

**Data Filename**	SWJ41.d	**Sample Name**	SWJ41
**Sample Type**	Sample	**Position**	P1-A5
**Instrument Name**	Instrument 1	**User Name**	
**Acq Method**	SIBU.m	**Acquired Time**	5/5/2015 3:09:39 PM
**IRM Calibration Status**	Success	**DA Method**	Default.m
**Comment**			

Sample Group **Info.**
Acquisition SW 6200 series TOF/6500 series
Version Q-TOF B.05.01 (B5125.2)

User Spectra

Fragmentor Voltage	Collision Energy	Ionization Mode
135	0	ESI

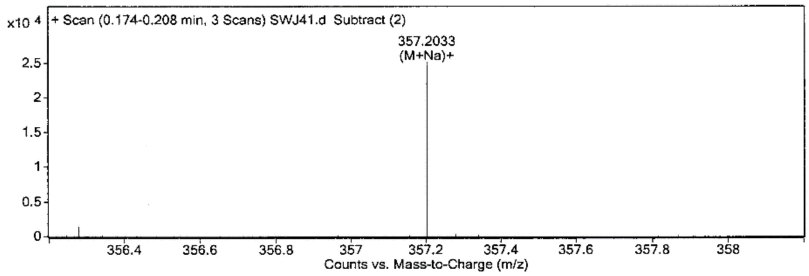

Peak List

m/z	z	Abund	Formula	Ion
197.1174		7147.94		
277.1296	1	6392.98		
279.1574	1	6469.99		
281.1884	1	9256.2		
299.2004	1	11599.76		
334.2376	1	35864.13		
335.2405	1	8276.27		
357.2033	1	25134.21	C20 H30 O4	(M+Na)+
373.1782	1	28991.58		
374.1806	1	5740.64		

Formula Calculator Element Limits

Element	Min	Max
C	3	60
H	0	120
O	0	30
N	0	10

Formula Calculator Results

Formula	CalculatedMass	CalculatedMz	Mz	Diff. (mDa)	Diff. (ppm)	DBE
C20 H30 O4	334.2144	357.2036	357.2033	-0.2	-0.7	6.0000

Optical rotation measurement

Model : P-1020 (A060460638)

No.	Sample	Mode	Data	Monitor<br>Blank	Temp.<br>Cell<br>Temp Point	Date<br>Comment<br>Sample Name	Light<br>Filter<br>Operator	Cycle Time<br>Integ Time
No.1	3 (1/3)	Sp.Rot	29.3420	0.0223<br>0.0000	24.8<br>50.00<br>Cell	Tue May 05 17:35:17 2015<br>0.00152g/mL MeOH<br>SWJ41	Na<br>589nm	2 sec<br>10 sec
No.2	3 (2/3)	Sp.Rot	29.7370	0.0226<br>0.0000	24.8<br>50.00<br>Cell	Tue May 05 17:35:30 2015<br>0.00152g/mL MeOH<br>SWJ41	Na<br>589nm	2 sec<br>10 sec
No.3	3 (3/3)	Sp.Rot	30.1320	0.0229<br>0.0000	24.7<br>50.00<br>Cell	Tue May 05 17:35:43 2015<br>0.00152g/mL MeOH<br>SWJ41	Na<br>589nm	2 sec<br>10 sec

+39.2368°

Figure S14. HR-ESI-MS and ORD spectra of enanderianin Q (**3**).

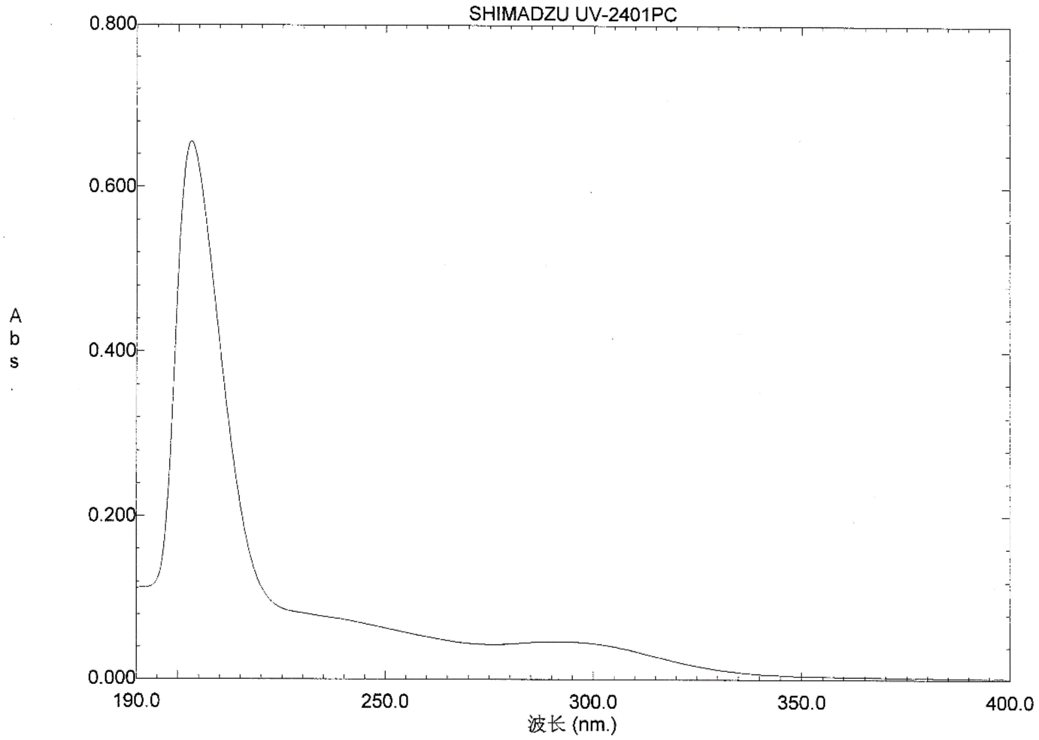

文件名: SWJ41

SWJ41 ———

样品浓度：0.0304毫克/毫升
溶剂：甲醇

创建于: 20:04 15-05-05
数据: 原始

测量模式: Abs.
扫描速度: 中速
狭缝: 5.0
采样间隔: 0.2

否.	波长 (nm.)	Abs.
1	292.20	0.0464
2	203.20	0.6556

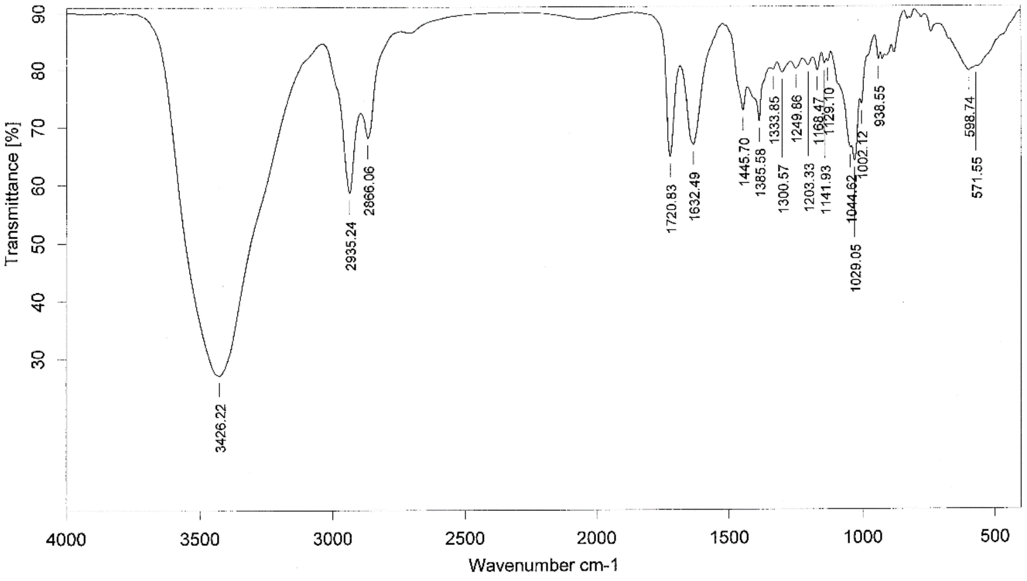

Sample : swj41	Frequency Range : 399.246 - 3996.32		Measured on : 08/05/2015
Technique : KBr压片	Resolution : 4	Instrument : Tensor27	Sample Scans : 16
Customer : 150508IR0	Zerofilling : 2	Acquisition : Double Sided,For	

Figure S15. UV (in MeOH) and IR (KBr) spectra of enanderianin Q (**3**).

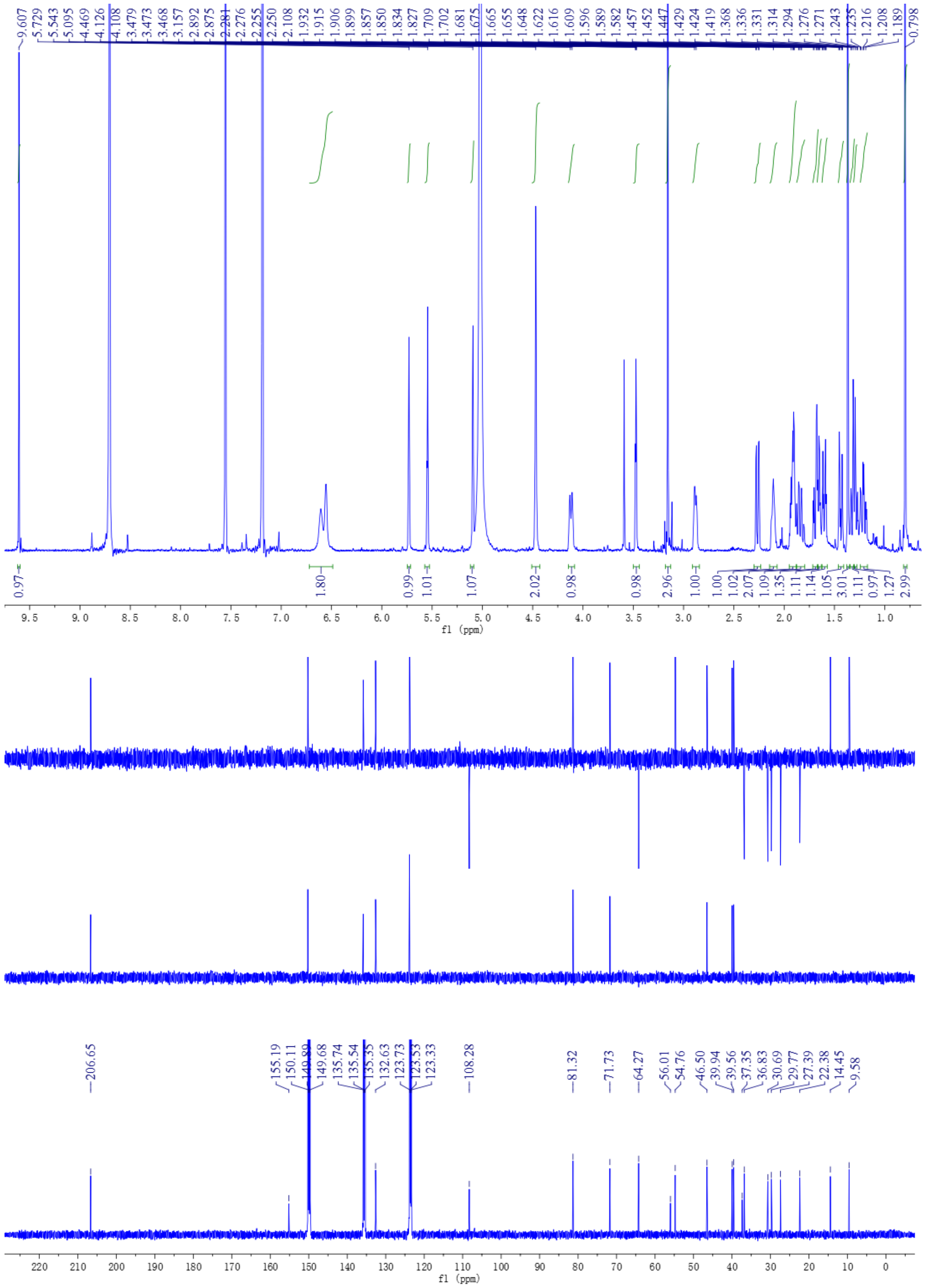

Figure S16. ^{1}H (500 MHz) and ^{13}C NMR (125 MHz) spectra of enanderianin R (**4**) in C_5D_5N.

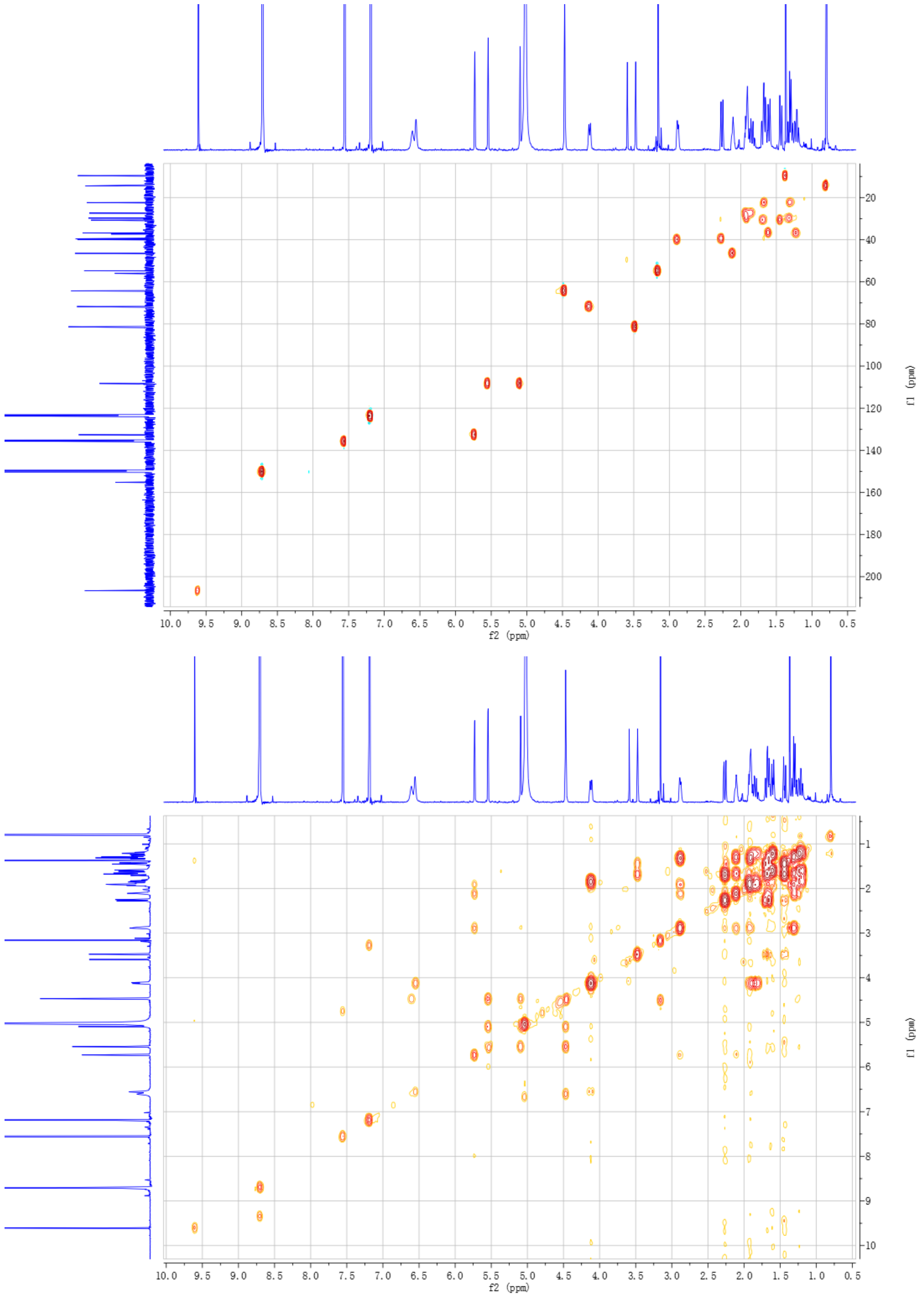

Figure S17. HSQC and ^{1}H-^{1}H COSY spectra of enanderianin R (**4**) in C_5D_5N (500 MHz).

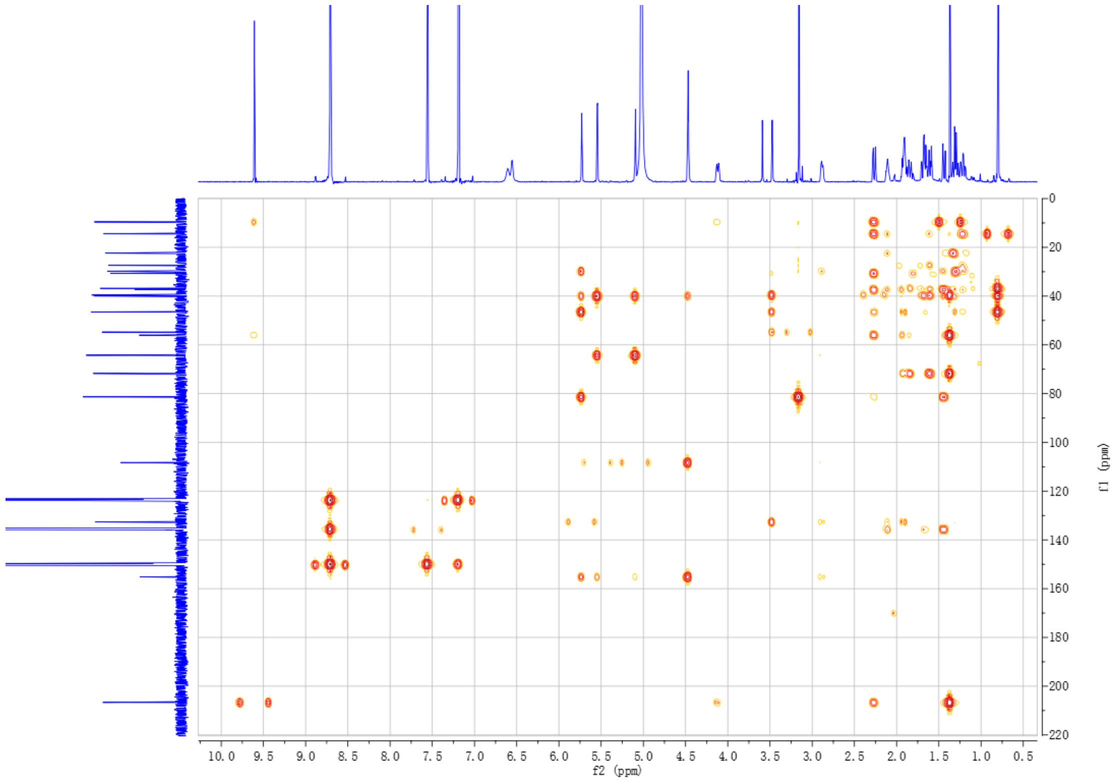

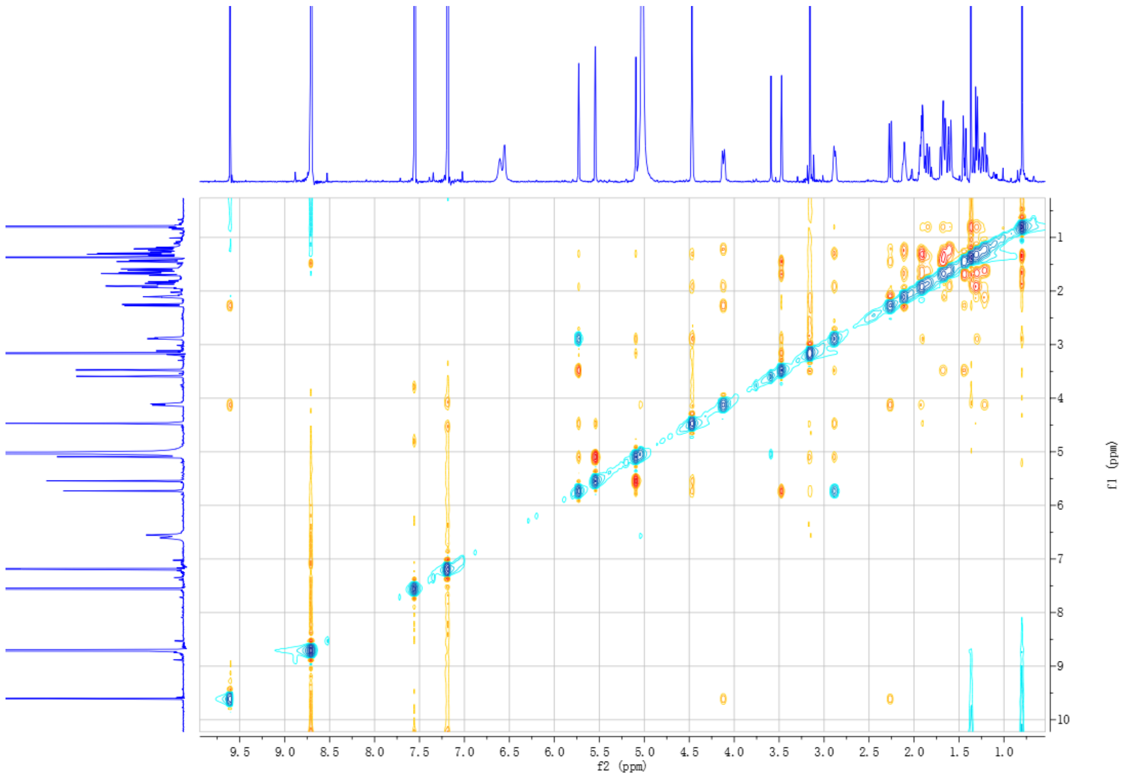

Figure S18. HMBC and ROESY spectra of enanderianin R (**4**) in C_5D_5N (600 MHz).

Qualitative Analysis Report

**Data Filename**	SWJ44.d	**Sample Name**	SWJ44
**Sample Type**	Sample	**Position**	P1-A6
**Instrument Name**	Instrument 1	**User Name**	
**Acq Method**	SIBU.m	**Acquired Time**	5/5/2015 3:11:21 PM
**IRM Calibration Status**	Success	**DA Method**	Default.m
**Comment**			

Sample Group **Info.**
Acquisition SW 6200 series TOF/6500 series
Version Q-TOF B.05.01 (B5125.2)

User Spectra

Fragmentor Voltage	Collision Energy	Ionization Mode
135	0	ESI

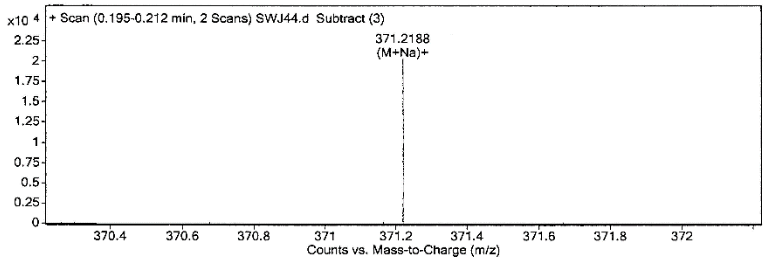

Peak List

m/z	z	Abund	Formula	Ion
157.0495	1	128023.12		
203.0941	1	48900.36		
277.1283	1	192300.58		
278.1317	1	26255.63		
294.1605	1	33786.62		
299.1106	1	124492.95		
315.0839	1	43561.67		
371.2188	1	20401.92	C21 H32 O4	(M+Na)+
387.193	1	30861.9		
408.2379	1	37039.21		

Formula Calculator Element Limits

Element	Min	Max
C	3	60
H	0	120
O	0	30
N	0	10

Formula Calculator Results

Formula	CalculatedMass	CalculatedMz	Mz	Diff. (mDa)	Diff. (ppm)	DBE
C21 H32 O4	348.2301	371.2193	371.2188	1.1	3.3	6.0000

Optical rotation measurement

Model : P-1020 (A060460638)

No.	Sample	Mode	Data	Monitor<br>Blank	Temp.<br>Cell<br>Temp Point	Date<br>Comment<br>Sample Name	Light<br>Filter<br>Operator	Cycle Time<br>Integ Time
No.1	18 (1/3)	Sp.Rot	-108.0000	-0.0054<br>0.0000	20.3<br>10.00<br>Cell	Fri Jan 13 13:21:51 2017<br>0.00050g/mL MeOH<br>SWJ44_1	Na<br>589nm	2 sec<br>2 sec
No.2	18 (2/3)	Sp.Rot	-100.0000	-0.0050<br>0.0000	20.3<br>10.00<br>Cell	Fri Jan 13 13:21:56 2017<br>0.00050g/mL MeOH<br>SWJ44_1	Na<br>589nm	2 sec<br>2 sec
No.3	18 (3/3)	Sp.Rot	-106.0000	-0.0053<br>0.0000	20.3<br>10.00<br>Cell	Fri Jan 13 13:22:02 2017<br>0.00050g/mL MeOH<br>SWJ44_1	Na<br>589nm	2 sec<br>2 sec
No.4	19 (1/3)	Sp.Rot	-106.0000	-0.0053<br>0.0000	20.3<br>10.00<br>Cell	Fri Jan 13 13:22:25 2017<br>0.00050g/mL MeOH<br>SWJ44_1	Na<br>589nm	2 sec<br>2 sec
No.5	19 (2/3)	Sp.Rot	-114.0000	-0.0057<br>0.0000	20.3<br>10.00<br>Cell	Fri Jan 13 13:22:30 2017<br>0.00050g/mL MeOH<br>SWJ44_1	Na<br>589nm	2 sec<br>2 sec
No.6	19 (3/3)	Sp.Rot	-100.0000	-0.0050<br>0.0000	20.3<br>10.00<br>Cell	Fri Jan 13 13:22:35 2017<br>0.00050g/mL MeOH<br>SWJ44_1	Na<br>589nm	2 sec<br>2 sec
No.7	20 (1/3)	Sp.Rot	-106.0000	-0.0053<br>0.0000	20.3<br>10.00<br>Cell	Fri Jan 13 13:23:17 2017<br>0.00050g/mL MeOH<br>SWJ44_1	Na<br>589nm	2 sec<br>2 sec
No.8	20 (2/3)	Sp.Rot	-94.0000	-0.0047<br>0.0000	20.3<br>10.00<br>Cell	Fri Jan 13 13:23:23 2017<br>0.00050g/mL MeOH<br>SWJ44_1	Na<br>589nm	2 sec<br>2 sec
No.9	20 (3/3)	Sp.Rot	-100.0000	-0.0050<br>0.0000	20.3<br>10.00<br>Cell	Fri Jan 13 13:23:28 2017<br>0.00050g/mL MeOH<br>SWJ44_1	Na<br>589nm	2 sec<br>2 sec

-103.7778°

Figure S19. HR-ESI-MS and ORD spectra of enanderianin R (**4**).

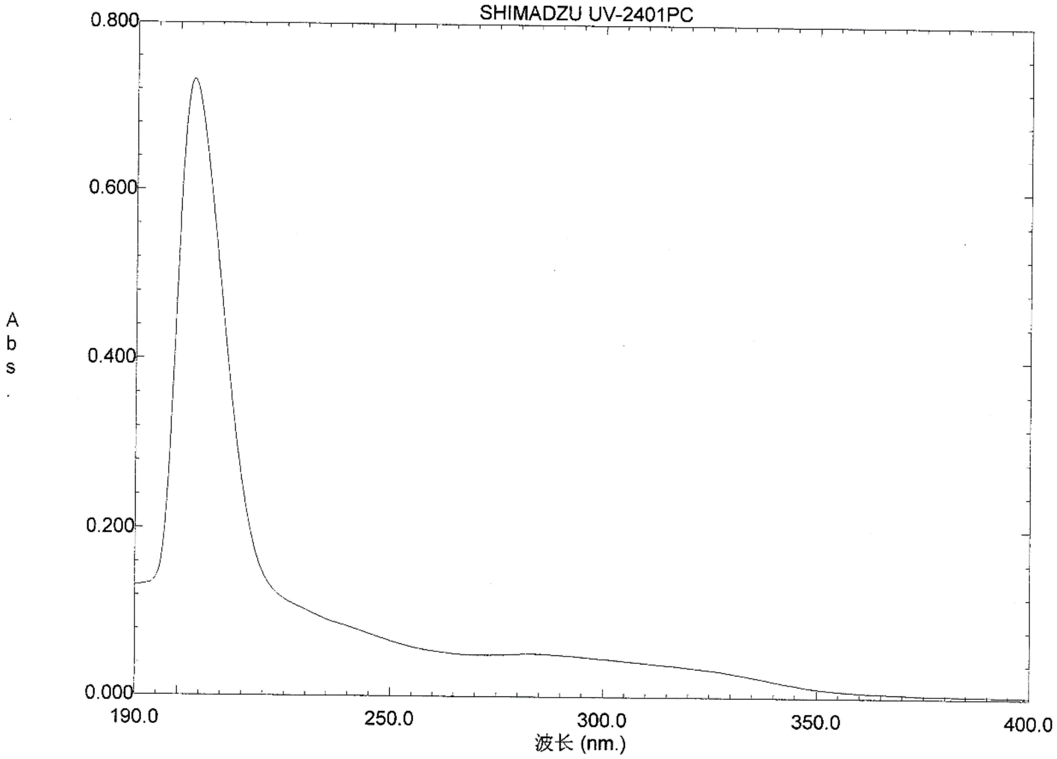

文件名: SWJ44

SWJ44 ———

创建于: 20:11 15-05-05
数据: 原始

样品浓度：0.0302毫克/毫升
溶剂：甲醇

测量模式: Abs.
扫描速度: 中速
狭缝: 5.0
采样间隔: 0.2

否.	波长 (nm.)	Abs.
1	283.40	0.0517
2	203.40	0.7318

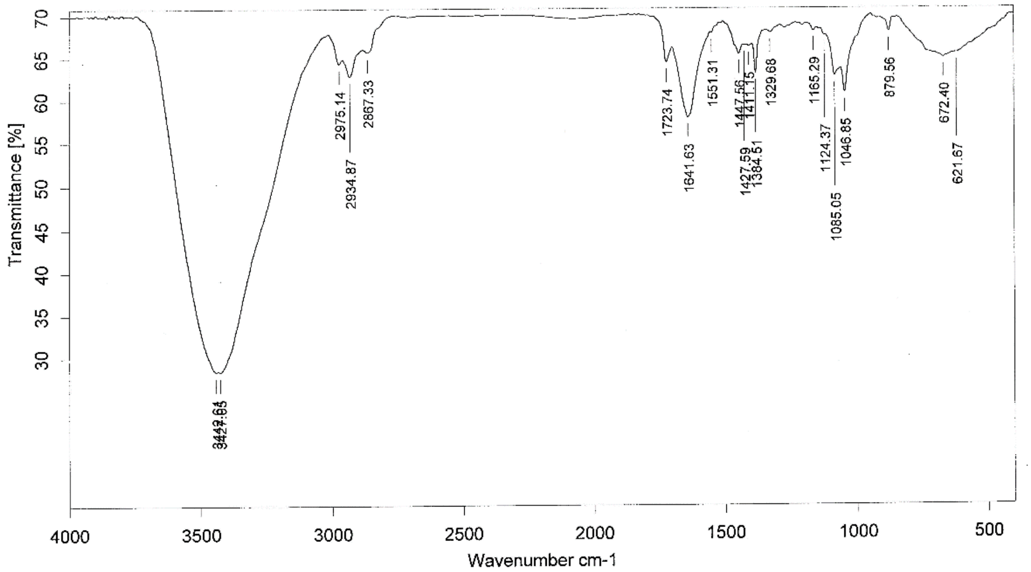

Sample : swj44	Frequency Range : 399.246 - 3996.32		Measured on : 08/05/2015
Technique : KBr压片	Resolution : 4	Instrument : Tensor27	Sample Scans : 16
Customer : 150508IR1	Zerofilling : 2	Acquisition : Double Sided,For	

Figure S20. UV (in MeOH) and IR (KBr) spectra of enanderianin R (**4**).
